# Highly Polygenic Control of Photosynthetic Responses to Nighttime Temperature Studied by Genomic Prediction

**DOI:** 10.1111/pce.70577

**Published:** 2026-05-05

**Authors:** Ana Carolina dos Santos Sá, Alina Bazarova, Andreas Fischbach, Stefan Kesselheim, Benjamin Stich, Shizue Matsubara

**Affiliations:** ^1^ IBG‐2: Plant Sciences, Forschungszentrum Jülich Jülich Germany; ^2^ Heinrich‐Heine‐Universität Düsseldorf Düsseldorf Germany; ^3^ Jülich Supercomputing Centre, Forschungszentrum Jülich Jülich Germany; ^4^ Institute of Quantitative Genetics and Genomics of Plants, Heinrich‐Heine‐Universität Düsseldorf Düsseldorf Germany; ^5^ Cluster of Excellence on Plant Sciences (CEPLAS) Germany; ^6^ Julius Kühn‐Institut, Institute for Breeding Research on Agricultural Crops Sanitz Germany; ^7^ Utilization of Plant Genetic Resources for Breeding Purposes University of Rostock Rostock Germany

**Keywords:** Arabidopsis, gBLUP, genomic prediction, GWAS, natural genetic variation, nighttime temperature, photosynthesis, random forest

## Abstract

Rising nighttime temperature (T_night_) can reduce crop yields while low T_night_ may restrict plant growth and development. Despite quantifiable effects of T_night_, genetic basis underlying plant responses to T_night_ remains unclear. We investigated natural variation in long‐term response of effective photosynthetic efficiency (F_q_’/F_m_’) to T_night_ among Arabidopsis accessions. Genome‐wide association study (GWAS) was conducted for F_q_’/F_m_’ of the plants grown under 15°C or 20°C T_night_. GWAS revealed highly polygenic architecture of F_q_’/F_m_’, with associated single nucleotide polymorphisms (SNPs) varying across T_night_ conditions and measurement days. Notably, 15°C T_night_ stabilised the contributions of a subset of SNPs, whereas 20°C T_night_ enhanced day‐to‐day variations in SNP‐trait associations. We then incorporated the associated SNPs in genomic prediction (GP) models to assess the improvement of prediction accuracy. The GWAS‐derived SNPs significantly improved the prediction ability of GP models, indicating collective influence of numerous small‐effect SNPs. Finally, the model predictions were experimentally validated in an independent, genetically diverse population, which confirmed the correct identification of low‐F_q_’/F_m_’ accessions in 15°C T_night_. These results uncover the genetic underpinnings of long‐term F_q_’/F_m_’ response to cool *vs* warm nights and establish a framework for leveraging GWAS and GP to explore complex traits, such as photosynthesis, toward breeding climate‐resilient crops.

## Summary statement

- Effective photosynthetic efficiency is a highly polygenic trait, with the associated genetic markers changing across nighttime temperature conditions and measurement days.
- Cooler nights stabilise the contributions of a subset of genetic associations while warmer nights enhance day‐to‐day variation.

## 1 Introduction

Global climate change is increasing nighttime temperature (T_night_) more rapidly than daytime temperature (T_day_) (Sillmann et al. 2013; Davy et al. 2017; Cox et al. 2020). Episodes of elevated T_night_ can cover larger geographic areas and last longer than those of high T_day_ (Sadok and Jagadish 2020). While heatwaves and maximal T_day_ have gained widespread attention in plant science and crop research, the impacts of nighttime warming are understudied. Yet, high T_night_ can reduce yields of staple crops such as rice (Peng et al. 2004; Bahuguna et al. 2017), wheat and barley (García et al. 2016) and pose a threat to agricultural sustainability and food security.

The mechanisms underlying yield losses at elevated T_night_ are complex, including altered carbon allocation due to increased nocturnal respiration (Bahuguna et al. 2017) and decreased sugar export from leaves (Tombesi et al. 2019), as well as a shorter grain‐filling period (García et al. 2016; Impa et al. 2019). Besides the direct effects of T_night_ on nocturnal carbon partitioning and utilisation, previous studies have highlighted (indirect) effects on daytime photosynthesis in different species (Prasad et al. 2008; Jing et al. 2016; Tombesi et al. 2019). A meta‐analysis indicated a small increase in net carbon assimilation rate in woody plants under high T_night_, but a slight decrease in herbaceous plants, including crops (Jing et al. 2016). Because elevated temperatures enhance respiration, high T_night_ may reduce net carbon gain and alter resource allocation in plants, particularly when photosynthesis does not meet the increased carbon demand.

Whilst high T_night_ may decrease crop yields by curtailing the allocation to reproductive organs, low T_night_ can limit plant growth and biomass accumulation (Jing et al. 2016). Growth suppression at low T_night_ is often accompanied by a decline in photosynthesis, even at moderately low T_night_ (Paul and Foyer 2001; Tombesi et al. 2019; Sadok and Jagadish 2020). Despite these quantifiable effects of T_night_ on growth, photosynthesis, and yields, the genetic underpinnings of plant responses to T_night_ remain poorly understood.

The analysis of natural genetic variation offers valuable insights into genetic architecture underlying a range of plant traits (Alonso‐Blanco et al. 2009). Genome‐wide association study (GWAS)–an approach that statistically tests for associations between genetic markers and phenotypic variation (Korte and Farlow 2013)–uncovers trait‐associated genetic variants and therewith functional polymorphisms. Although GWAS has proven powerful in identifying allelic variants associated with important agricultural traits, unravelling the genetic architecture of polygenic traits, which are governed by numerous small‐effect variants, remains a major challenge (Korte and Farlow 2013). Meanwhile, a paradigm shift has occurred in the study of complex traits, from identifying genetic variants with strong statistical associations to developing accurate prediction models (de los Campos et al. 2018).

Genomic prediction (GP) builds a model using genome‐wide markers and phenotypic data to estimate breeding values for genotypes with marker information alone (Meuwissen et al. 2001). Genomic best linear unbiased prediction (gBLUP) has been widely used in plant and animal breeding (VanRaden 2008; Crossa et al. 2017); however, because of its additive nature, alternative approaches have been developed to capture complex, non‐additive interactions among markers, such as epistasis (Pérez‐Rodríguez et al. 2012; Azodi et al. 2019). Examples of non‐linear models include ensemble machine learning methods which combine multiple weak models into a single, more robust and accurate model (Ren et al. 2016). Whether non‐linear models can consistently surpass linear models in predicting complex polygenic traits remains an unsettled question in quantitative genetics (Pérez‐Rodríguez et al. 2012; Azodi et al. 2019).

Here, we explored natural genetic variation underlying long‐term photosynthetic responses to two T_night_ conditions (15°C and 20°C) in *Arabidopsis thaliana*. Our GWAS revealed numerous and distinct single nucleotide polymorphisms (SNPs) associated with variations in effective photosystem II (PSII) efficiency (F_q_’/F_m_’) among the accessions grown under 15°C and 20°C T_night_. We used F_q_’/F_m_’ as a proxy measure of photosynthetic performance because it correlates with the quantum yield of CO_2_ assimilation under laboratory conditions (Genty et al. 1989). Moreover, PSII quantum yields can be measured rapidly and non‐invasively, making them well suited for high‐throughput time‐course experiments involving many samples. Midday F_q_’/F_m_’ showed a highly polygenic architecture, with hundreds of small‐effect SNPs contributing variably across different days and T_night_ conditions. Incorporating these GWAS‐derived SNPs into linear and non‐linear GP models markedly improved prediction accuracy in both T_night_ conditions, highlighting their collective influence on F_q_’/F_m_’. Remarkably, 15°C T_night_ stabilised the contributions of a subset of associated SNPs, whereas 20°C T_night_ induced larger day‐to‐day variation in SNP‐trait associations. A validation experiment confirmed the models’ capacity to identify low‐F_q_’/F_m_’ accessions in an independent, genetically diverse population, demonstrating the potential of this approach for advancing our understanding and prediction of complex traits.

## 2 Materials and Methods

### 2.1 Plant Materials and Growth Conditions

The accessions of *Arabidopsis thaliana* (L.) Heynh. (Supporting Information S1: Table S1) from the global HapMap population (Li et al. 2010) were purchased from the Nottingham Arabidopsis Stock Centre (N76309). Seeds were sown automatically in 576‐cell trays containing moist substrate (type Topf 1.5; Balster Einheitserdewerk, Fröndenberg, Germany) with the automated PhenoSeeder system (Jahnke et al. 2016). After 3 to 4 days of stratification at 4°C in the dark, the trays with seeds were transferred to a climate chamber running with 12 h/12 h light/dark cycle at 26°C T_day_, 20°C or 15°C T_night_, and constant 60% relative air humidity. The illumination was provided by white LEDs (XLamp CXA2520; Cree LED, Durham, NC, USA). The diurnal light intensity regime was as follows: 4 h of linear increase to the maximum intensity of ~600 µmol photons m^−2^ s^−1^ (measured at the plant height), then 4 h of constant light at this intensity, followed by 4 h of linear decrease. The daily light integral was ~18.7 mol photons m^−2^ d^−1^.

Germination was monitored daily at around midday (4.5−7.5 h into the light period) for 8 days after stratification (DAS) using an automated image‐based device (Scharr 2020). On 11 DAS, seedlings were transferred to pots (7 × 7 × 7 cm; one plant per pot) filled with substrate (Lignostrat Dachgarten extensiv; HAWITA, Vechta, Germany). Seedlings were carefully transferred with the soil in which they were growing, ensuring that roots remained in the soil and shoots were not touched throughout the process. The pots were subsequently placed under the aforementioned conditions in the climate chamber. They were randomly placed in plastic inlets (24 pots per inlet; Nr.68124; Bachmann Plantec, Hochdorf, Switzerland) where they stayed throughout the experiments (Supporting Information S1: Figure S1). Accession*treatment*replication combinations were evaluated in six independent experiments (three experiments per treatment), with Col‐0 included as reference in each experiment to check the variability among replicate plants across the three experiments. Plants were well‐watered throughout cultivation and experiments.

An additional experiment was conducted using nine selected accessions (Bs‐1, CUR‐8, DralIV3‐8, Ei‐2, Kyl‐1, LL‐0, PAR‐8, Paw‐4 and Sim‐1; Supporting Information S1: Table S2) and Col‐0 (as reference) to validate the model prediction of F_q_’/F_m_’ in an independent set of accessions. Seeds of these accessions were purchased from the Nottingham Arabidopsis Stock Centre and treated in the same way as described above. Plants were placed in the plastic inlets under the 15°C T_night_ conditions. The number of replicate plants used in the validation experiment was: Bs‐1, *n* = 14; Col‐0, *n* = 15; CUR‐8, *n* = 19; DraIV3‐8, *n* = 18; Ei‐2, *n* = 3; Kyl‐1, *n* = 4; LL‐0, *n* = 27; PAR‐8, *n* = 4; Paw‐4, *n* = 13; Sim‐1, *n* = 11.

### 2.2 Rosette Growth Analysis

Projected rosette area was measured on 16, 17, 18 and 19 DAS using the image‐based GROWSCREEN method (Jansen et al. 2009). The measurements were performed at around midday (4.5−8 h into the light period) when leaves were positioned almost horizontally. Relative growth rate (RGR, % d^−1^) was calculated for each plant using the projected rosette area measured on two consecutive days, as previously described (Meyer et al. 2021).

### 2.3 Chlorophyll *a* Fluorescence Analysis

Chlorophyll *a* fluorescence measurements were performed in dark‐adapted and light‐adapted plants under the growth conditions in the climate chamber on 16, 17 and 18 DAS. Dark‐adapted plants were measured during the night (8−11.5 h into the dark period) at the respective T_night_ (20°C or 15°C). Light‐adapted plants were measured around midday (4.5−8 h into the light period) when the light intensity stayed at ca. 600 μmol photons m^−2^ s^−1^ and the air temperature was 26°C. Measurements were performed using a light‐induced fluorescence transient (LIFT) instrument (model LIFT‐REM 1.0; Soliense Inc., Shoreham, New York, USA) mounted on a tripod (Advanced VX mount; Celestron, Torrance, USA) with an automated positioning system. The plastic inlets with 24 plants were placed one by one in the designated position in front of the instrument (Supporting Information S1: Figure S1). The distance between the instrument and the plants was between 63.5 and 71.8 cm, depending on the position of the plants inside the inlet, and remained constant for each plant throughout the experiment. The LIFT instrument was calibrated for measurements at a 60‐cm distance prior to the experiments.

We used the LIFT excitation protocol FRRF_0.75ms_ (Keller et al. 2019) which is based on the fast repetition rate approach (Kolber et al. 1998). This protocol employs an excitation flash consisting of 300 sub‐saturating short (1,6 μs) pulses (“flashlets”) triggered at a 2.5‐μs interval to reduce the primary quinone acceptor of PSII (Q_A_) for 0.75 ms, followed by 125 flashlets triggered at exponentially increasing intervals to monitor Q_A_ reoxidation for 209 ms. The excitation flashes were applied by four LED channels (445, 470, 505, and 535 nm) with the average intensity in the Q_A_ reduction phase of 21,890 and 18,050 µmol photons m^−2^ s^−1^ (determined according to Zendonadi dos Santos et al. (2021) at a distance of 60 cm from the LIFT instrument), respectively, in the GWAS experiments and the validation experiment. Emission of chlorophyll *a* fluorescence was detected at 685 (±10) nm. Measurements in dark‐adapted plants were performed with a single flash, while in light‐adapted plants five flashes were applied with a 1.5‐s interval to obtain a mean value. The light‐adapted measurements were started at least 30 s after placing the inlet in front of the LIFT.

The minimal (F_o_), the maximal (F_m_ and F_m_’) and steady‐state (F_s_) fluorescence yields measured by the LIFT protocol (Zendonadi dos Santos et al. 2021) were used to calculate the maximal (F_v_/F_m_ = (F_m_ – F_o_)/F_m_) and effective quantum yield of PSII (F_q_’/F_m_’ = (F_m_’ – F_s_)/F_m_’) in the dark‐adapted and light‐adapted states, respectively. We confirmed the stability of F_q_’/F_m_’ in Col−0 within the midday measurement window to ensure that phenotypic variations observed among the accessions were not confounded by time‐dependent changes in F_q_’/F_m_’.

### 2.4 Estimation of Adjusted Entry Means

Linear mixed models were used to obtain adjusted entry means and to assess the accessions’ performance without the variability introduced by confounding factors. Chlorophyll fluorescence and growth data were discarded when (i) plants exhibited severely impaired growth, (ii) plants were damaged by handling during the experiments, (iii) accessions had rosette architecture that interfered with 2D image analysis, (iv) accessions had only one replicate plant, or (v) plants were classified as outliers in the statistical analyses, that is, their inclusion in linear mixed models violated the model assumptions (normal distribution of residuals and homogeneity of variance) based on visual inspection. Plants identified as outliers on any single day within each T_night_ condition were removed from the entire dataset to ensure that the same individuals were evaluated across the three measurement days (16–18 DAS). One to two replicates per accession per experiment remained for both 15°C and 20°C T_night_ after data cleaning, yielding a total of two to five replicates per accession per treatment across the 3 days; for some accessions, data from one experiment were discarded. For the reference accession Col‐0, two to five replicates were included per experiment, resulting in six and ten total replicates for 15°C and 20°C T_night_, respectively. After cleaning, phenotypic data from 308 accessions remained for further analyses.

Linear mixed models were fitted to the phenotypic data to account for the spatial heterogeneity of growth and LIFT measurement conditions (Supporting Information S1: Figure S1) along with other random factors such as germination date. When the distribution and variance of residuals violated the assumptions of linear mixed models, the response variable was transformed using either a logarithmic or a power transformation (raised to the seventh or the 30th power). The models used were:

Model 1: Adjust entry means of projected rosette area

*log (rosette area) = accession + germination date + experiment + experiment:accession + experiment:plant replicate* + *x position* + *y position* + *ε*

Model 2: Adjust entry means of rosette RGR

*RGR = accession + germination date + experiment + experiment:accession + experiment:plant replicate* + *x position* + *y position* + *ε*

Model 3: Adjust entry means of F_v_/F_m_

*F*_*v*_ */F*_*m*_^^30^ *= accession + germination date + experiment + experiment:plant replicate* + *x position* + *y position* + *LIFT* + *ε*

Model 4: Adjust entry means of F_q_’/F_m_’

*F*_*q*_ *’/F*_*m*_ *’* ^^7^ *= accession + germination date + experiment + experiment:plant replicate* + *x position* + *y position* + *LIFT* + *ε*

where the response variables were modelled as a function of the day on which seedlings germinated (i.e., germination date), experiment number (#1 to #3 for each T_night_ treatment), plant replicate nested (indicated by colon in the above equations) in each experiment, x and y position within the inlet (accounting for variations in growth conditions; Supporting Information S1: Figure S1), and plant position during the LIFT measurements (*LIFT*; accounting for variations in the distance and angle between the LIFT instrument and plants during the measurements; Supporting Information S1: Figure S1). The term *ε* represents the residual of the model, that is, variation in the response variable that is not explained by the factors included in the linear mixed model. The effect of accessions was fixed, while germination date, experiment, accession nested in experiment, plant replicate nested in experiment, x–y positions, and LIFT measurement positions were random. Adjusted entry means of F_q_’/F_m_’ across three days combined in the 15°C T_night_ condition were obtained by using Model 4 with day as an additional random effect. The model used for the validation experiment with 10 accessions was:

Model 5: Adjust entry means of F_q_’/F_m_’

*F*_*q*_ *’/F*_*m*_ *’ = accession + germination date + plant replicate* + *x position* + *y position* + *LIFT* + *ε*

The models were fitted separately for each T_night_ treatment and measurement day using the *lmer* function in the *lme4* package of R (version 4.2.2) (R Core Team 2022). Adjusted entry means were obtained using the function *emmeans* in the *emmeans* package. The model assumptions (i.e., normal distribution of residuals and homogeneity of variance) were verified for each model. Statistically significant differences between the T_night_ treatments were tested by two‐tailed Student *t*‐test (equal or unequal variance checked by F‐test) using the *t. test* function. Broad‐sense heritability, defined as the proportion of genetic variance to phenotypic variance, was estimated separately for each T_night_ condition and measurement day according to Piepho and Möhring (2007) using Models 1–4 with accession as random effect.

### 2.5 Genome‐Wide Association

Of the 308 accessions in the phenotyping experiments (Supporting Information S1: Table S1), 15 (627ME‐4Y1, BUI, CLE‐6, DraIV1‐7, Gul1‐2, Kas‐2, NC‐6, PHW‐34, PUZ24, RRS, Sav‐1, TOU‐A1‐12, TOU‐A1‐67, UKCW06202 and Wil‐1) lacked SNP data and, thus, were excluded from GWAS. The SNP information was obtained from the regional mapping panel (RegMap; Horton et al. 2012; method 75, accessed on 23.03.2023). The SNP dataset contained 214,051 variants, which were reduced to 211,771 variants after filtering out minor allele frequency (< 2%) using PLINK (version v1.90b6.21) (Purcell et al. 2007). The ID numbers of the accessions (listed in Supporting Information S1: Table S1) were obtained from https://bergelsonlab.org/resources/a-thaliana/ (accessed on 22.03.2023). When the ID numbers were inconsistent between the SNP dataset and the accession list (Bg‐2, Br‐0, Col‐0, Got‐7 and Oy‐0), information was obtained from http://naturalsystems.uchicago.edu/naturalvariation/hapmap/ (accessed on 23.03.2023).

The analysis of SNP‐trait association was performed separately for each T_night_ condition and measurement day by GAPIT (version 3) using FarmCPU model (Liu et al. 2016) based on the adjusted entry means of F_q_’/F_m_’. The F_q_’/F_m_’ data of 293 accessions were included in the GWAS on DAS 17 and 18, whereas one accession (T1080) was excluded on DAS 16. Population structure was accounted for by using principal component (PC). To select an appropriate number of PC, we performed PC analysis based on the 211,771 SNPs (Supporting Information S1: Figure S2) and fitted the linear mixed models to F_q_’/F_m_’ by adding an increasing number of PC as covariates with the *sommer* package. Up to five PCs that most explained the variance in F_q_’/F_m_’, assessed as the correlation coefficient based on log likelihood (Magee and Magee 1990), were included in the FarmCPU (Supporting Information S1: Figure S3). Gene annotation was retrieved from TAIR10 (https://www.arabidopsis.org/).

The proportion of phenotypic variance explained by individual SNPs or sets of SNPs was estimated by fitting the linear mixed models with SNPs as fixed effects using the *lm* function in the *stats* package of R. When pairs of SNPs were in linkage disequilibrium (*R* ^2^ > 0.8), only one of them was included in a set of SNPs (simultaneous fit). The proportion of explained phenotypic variance was assessed as the squared correlation coefficient (*r* ^2^) between the actual and predicted F_q_’/F_m_’ in five‐fold cross validation using 40 replications. Effects of the SNP‐trait associations detected in the full dataset were estimated newly in each run of the cross validation. For this purpose, phenotypic data were randomly split into five groups, of which four comprised the training set and the remaining group served as the test set. The models were then trained on the training set and evaluated on the test set. This procedure was repeated five times using each of the five groups as the test set.

### 2.6 Genomic Prediction

Genomic prediction of F_q_’/F_m_’ was performed using gBLUP (VanRaden 2008) and two ensemble machine learning methods, namely random forest (Ho 1995; Breiman 2001) and eXtreme Gradient Boosting (XGBoost; Chen and Guestrin 2016). We additionally tested Bayesian models BayesA and BayesB (Meuwissen et al. 2001) to verify whether prediction ability based on the 211,771 SNPs can be improved by assuming non‐homogenous effects of SNPs. Prediction models were fitted to the adjusted entry means of F_q_’/F_m_’ separately for each T_night_ treatment and measurement day. The F_q_’/F_m_’ data of all 293 accessions included in the GWAS were used for training and testing the models on 17 and 18 DAS, whereas one accession (T1080) was excluded on 16 DAS. Three different SNP sets were used as predictors: (i) all 211,771 SNPs used in the GWAS, (ii) GWAS‐derived SNPs with ‐log_10_ (*P*) ≥ FDR (false discovery rate) threshold (0.05) and/or ‐log_10_ (*P*) > 4.0 on at least two measurement days, and (iii) GWAS‐derived SNPs with ‐log_10_ (*P*) ≥ 3.0 on each day.

Genomic prediction based on gBLUP was performed with the *sommer* package of R. Prediction ability was estimated as the median Pearson correlation coefficient between the actual and the predicted F_q_’/F_m_’ across all five‐fold cross validation runs. BayesA and BayesB were used as implemented in the *BGLR* package (Pérez and de los Campos 2014) based on the 211,771 SNPs. Gibbs sampling was performed with 30,000 iterations, of which 10,000 samples were discarded as burn‐in. Prediction using random forest was based on GWAS‐derived SNPs, namely, the ones with ‐log_10_ (*P*) ≥ FDR (0.05) and/or ‐log_10_ (*P*) > 4.0 on at least two measurement days, and those with ‐log_10_ (*P*) ≥ 3.0 on each day. The prediction was performed by the *caret* package (Kuhn 2008) of R using 500 trees and fivefold cross validation. The number of features selected as predictors in each tree node was *p*−1, where *p* is the total number of SNPs in the set.

Genomic prediction by XGBoost based on the 211,771 SNPs was performed on the JUWELS‐Booster high‐performance computing system (https://apps.fz-juelich.de/jsc/hps/juwels/booster-overview.html) of Forschungszentrum Jülich (Kesselheim 2021). We used XGBoost as part of the Scikit‐Learn library (Pedregosa 2011) in Python 3.10.4 (https://www.python.org). To identify the combination of hyperparameters that maximised the predictive performance of XGBoost, we performed a grid search (Supporting Information S1: Table S3) with mean squared error as the loss function. Since the grid search over maximal depth of tree and number of estimators yielded comparable predictive performance, the prediction results are presented for the grid search over maximal depth of tree (3 or 5) and 100 trees. Correction for population structure was performed by taking the residuals of 250 PCs from a PC analysis of the genotypes (Zhao et al. 2012) as the inputs for prediction by XGBoost. Since similar prediction of F_q_’/F_m_’ was obtained with 100 or 500 trees and with 3, 5, 6 or 10 maximum tree depth as described above, grid search was performed over the maximum depth (3 or 5) with 100 estimators.

The predictive performance of gBLUP and random forest (based on the GWAS‐derived SNPs) or gBLUP and XGBoost (211,771 SNPs) was compared by using the same data sets for training as well as for testing. Phenotypic data of the 293 accessions were randomly split into training and test sets at 4:1 ratio and three independent replicates were run. Predictive performance was assessed as the prediction ability (as described above) and root mean square error (RMSE) of the test sets. The F_q_’/F_m_’ data of all 293 accessions were used for training and testing the models on 17 and 18 DAS, whereas one accession (T1080) was excluded on 16 DAS.

The prediction models trained and tested on F_q_’/F_m_’ of the 293 accessions were used to predict F_q_’/F_m_’ for 1.014 genetically diverse, non‐phenotyped accessions from the RegMap population (Horton et al. 2012) using gBLUP and random forest based on the GWAS‐derived SNPs with ‐log_10_ (*P*) ≥ 3.0. Nine accessions having low, intermediate, and high predicted F_q_’/F_m_’ values were selected (Supporting Information S1: Table S2) and used for experimental validation of the model prediction in 15°C T_night_. Col‐0 was included as reference in the validation experiment.

## 3 Results

### 3.1 Natural Genetic Variation in Long‐Term Response of Photosynthesis to T_night_

To assess genetic diversity in long‐term photosynthetic response to T_night_, we measured PSII efficiency in 308 Arabidopsis accessions (Supporting Information S1: Table S1) from the HapMap population (Li et al. 2010) grown in 15°C and 20°C T_night_ condition. Both T_night_ treatments had the same T_day_ of 26°C. Col‐0 plants were included as reference in every experiment to assess the variability among plant replicates across the three experiments for each T_night_ condition. The total number of Col‐0 replicates was six for 15°C T_night_ and ten for 20°C T_night_. The PSII efficiency of Col‐0 was highly reproducible across the experiments, as indicated by the small standard deviations (SDs) around the observed (non‐adjusted) means (0.45–0.48 ± 0.009–0.021 for F_q_’/F_m_’, 0.77–0.79 ± 0.003–0.011 for F_v_/F_m_; Supporting Information S1: Table S4). In comparison, growth‐related traits, particularly projected rosette area, showed greater variability among replicates across the experiments.

**Figure 1:**
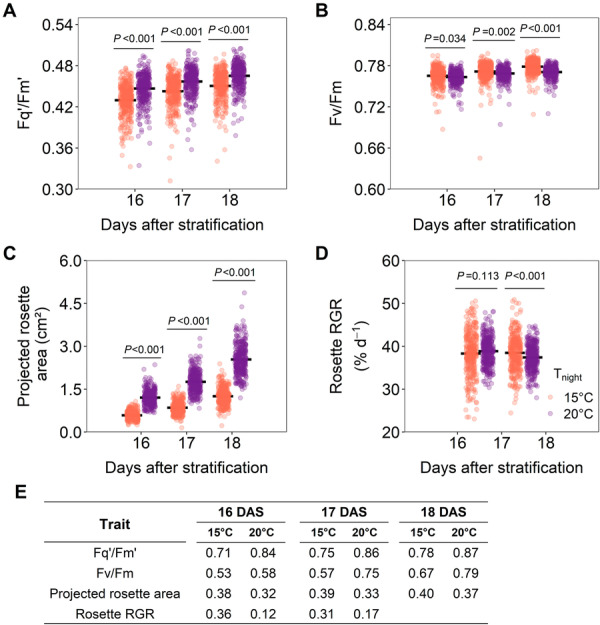
Effects of night temperature (T_night_) on PSII efficiency and growth traits. (A, B) The effective (F_q_’/F_m_’) and the maximal (F_v_/F_m_) PSII efficiency. (C, D) Projected rosette area and relative growth rate (RGR). (E) Broad‐sense heritability of these traits. Traits were measured in 308 Arabidopsis accessions from the HapMap population (listed in Supporting Information S1: Table S1) grown in the 15°C (coral) and 20°C (purple) T_night_ conditions on 16, 17 and 18 days after stratification (DAS). F_q_’/F_m_’ was measured at around midday under the maximal growth light intensity of ~600 μmol photons m^−2^ s^−1^ and daytime growth temperature of 26°C, while F_v_/F_m_ was measured at respective T_night_ during the night. The rosette RGR was calculated from the projected rosette area measured on two consecutive days (i.e., RGR on 16 DAS was calculated from the projected rosette area on 16 and 17 DAS). Horizontal lines in panel A–D show means of all accessions. Circles represent adjusted entry means of individual accessions (*n* = 2–10 for each accession). Significant differences between the two treatments were tested by two‐tailed *t*‐test.

The adjusted entry means of midday F_q_’/F_m_’ under the maximal growth light intensity (~600 μmol photons m^−2^ s^−1^) were, on average across all accessions, significantly (α = 0.05) higher in the plants grown in 20°C compared with 15°C T_night_ (Figure 1A). The opposite was the case for F_v_/F_m_ measured at the respective T_night_ during the night, with slightly but significantly (α = 0.05) higher values found for the plants grown in 15°C T_night_ (Figure 1B). The accessions showed large phenotypic variance for PSII efficiency in both T_night_ conditions, with larger variations for light‐adapted midday F_q_’/F_m_’ than for dark‐adapted F_v_/F_m_ (Figure 1A,B; Supporting Information S1: Table S5). Broad‐sense heritability (defined as the proportion of genetic variance to phenotypic variance) of the PSII efficiency parameters was generally high in our experimental conditions (Figure 1E), especially for F_q_’/F_m_’. The heritability of these parameters was higher in 20°C than in 15°C T_night_ and increased in both conditions from 16 to 18 DAS (Figure 1E).

Temperature has a strong impact on plant growth. Arabidopsis plants were smaller in 15°C than in 20°C T_night_ (Figure 1C). Compared with the cumulative effects of T_night_ on projected rosette area, the impact on daily RGR was less pronounced (Figure 1D), paralleling the small differences in midday PSII efficiency observed between 15°C and 20°C T_night_ (Figure 1A). While the accessions displayed large phenotypic variance also for the growth parameters, their heritability was much lower than that of PSII efficiency (Figure 1E).

The variability among plant replicates per accession was, on average across all accessions, smaller for the photosynthetic traits than for the growth traits, as indicated by SDs of the adjusted entry means (Supporting Information S1: Table S6). Because the linear mixed models considered random variations in the phenotypes caused by confounding factors, SDs of models’ residuals (*ε* in the model equations 1–4) per accession and averaged across all accessions were smaller than the respective SDs of the adjusted entry means (Supporting Information S1: Table S6), indicating that the models explained the data well and decreased the variability among plant replicates. Although model performance was rather poor for rosette RGR compared with the other traits (corresponding to the low heritability of RGR in Figure 1E), the models still reduced the variability among plant replicates per accession.

### 3.2 T_night_ Affects Genetic Architecture of F_q_’/F_m_’

Having seen the large phenotypic variance and high heritability of PSII efficiency in the plants grown in 15°C and 20°C T_night_, we conducted GWAS to map genetic variants underlying the natural variation in midday F_q_’/F_m_’ under these T_night_ conditions. Of the 308 accessions that were phenotyped, 293 were included in GWAS since SNP information was not available for the remaining 15 accessions.

**Figure 2:**
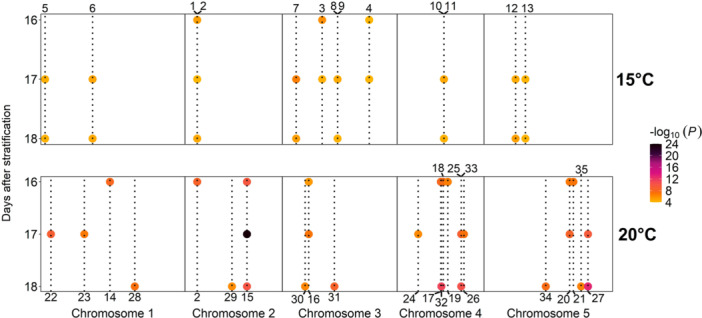
Summary of single nucleotide polymorphisms (SNPs) associated with long‐term acclimation of F_q_’/F_m_’ to night temperature. Genome‐wide SNP‐trait association was analyzed based on F_q_’/F_m_’ measured in the 293 Arabidopsis accessions grown in 15°C and 20°C T_night_ on 16, 17 and 18 days after stratification. SNP‐trait associations were considered significant when ‐log_10_ (*P*) ≥ FDR (false discovery rate) threshold (0.05) and/or ‐log_10_ (*P*) > 4.0 on at least two days. Circles represent individual SNPs. Colours of the circles show significance of association (*P*). SNPs are numbered from No. 1 to No. 13 for 15°C T_night_ and from No. 14 to No. 35 for 20°C T_night_. Information on these SNPs is provided in Supporting Information S1: Table S7. [Color figure can be viewed at wileyonlinelibrary.com]

Principal component analysis was performed for the 293 accessions based on 211,771 SNPs, excluding rare SNPs with an allele frequency below 2%. PC1 and PC2 explained 3.1% and 2%, respectively, of the total genetic variance among these accessions (Supporting Information S1: Figure S2). Up to five PCs were included in the GWAS models as covariates to correct for population structure (see Q‐Q plots in Supporting Information S1: Figure S3). The SNP‐trait associations identified by the model FarmCPU were considered significant when ‐log_10_ (*P*) ≥ FDR threshold (0.05) and/or ‐log_10_ (*P*) > 4.0 on at least 2 days (Supporting Information S1: Figure S4; Table S7). No more than 13 SNPs met the latter criterion in 15°C T_night_, of which SNP No. 2 was identified on all 3 days (Figure 2). None of these SNPs met the former criterion in 15°C T_night_. In 20°C T_night_, 23 SNPs met the former criterion, of which four (No. 15, 16, 20 and 27) also met the latter and No. 15 was identified on all 3 days (Figure 2). Except SNP No. 2 which was detected in both 15°C and 20°C T_night_, the SNP‐trait associations did not overlap between the two conditions.

The identified SNPs explained together 18%–32% of the F_q_’/F_m_’ variance in 15°C T_night_ and 40%–46% in 20°C T_night_ on different days (see r^2^ of simultaneous fits in Supporting Information S1: Table S7). Contributions of individual SNPs ranged from 5% to 12% in 15°C T_night_ and from 2% to 19% in 20°C T_night_, with the highest r^2^ found for SNP No. 7 in 15°C T_night_ and SNP No. 15 in 20°C T_night_ on 17 DAS. These SNPs also had the largest modulus of the effect (given in the unit of F_q_’/F_m_’), −0.022 for No. 7 and −0.024 for No. 15 (Supporting Information S1: Table S7). Most of the other SNPs had estimated effect size of ± 0.01 or smaller.

None of the identified SNPs is located in loci known to be directly involved in photosynthesis despite their association with F_q_’/F_m_’ (Supporting Information S1: Table S7). Of the aforementioned SNPs, for instance, SNP No. 2 is found in AT2G03730 encoding an ACT domain containing protein (ACT DOMAIN REPEAT 5), No. 7 in AT3G05050 encoding a protein kinase superfamily protein, and No. 15 in AT2G26250 encoding an enzyme 3‐ketoacyl‐CoA synthase (3‐KETOACYL‐COA SYNTHASE 10, aka FIDDLEHEAD) involved in the biosynthesis of very‐long‐chain fatty acids in epidermis and phloem (Pruitt et al. 2000).

### 3.3 GWAS‐Derived SNP‐Trait Associations Improve GP

If the variants identified by GWAS contribute to the genetic variance in F_q_’/F_m_’ among the accessions, information on these SNPs should enhance the power of GP to predict F_q_’/F_m_’ in these genotypes under the treatment conditions. Thus, to corroborate the collective influence of the GWAS‐derived SNPs on F_q_’/F_m_’, we evaluated the performance of gBLUP by using three different sets of SNPs as predictors: i) 211,771 SNPs in the Arabidopsis GWAS panel, ii) GWAS‐derived SNPs with ‐log_10_ (*P*) ≥ FDR threshold (0.05) and/or ‐log_10_ (*P*) > 4.0 on at least 2 days (Supporting Information S1: Table S7), and iii) GWAS‐derived SNPs with ‐log_10_ (*P*) ≥ 3.0 on each day (Supporting Information S2). The first set is for prediction without the information on GWAS‐derived SNP‐trait associations. The third set is based on a liberal significance threshold but contains more information on SNP‐trait associations compared to the second set (Supporting Information S1: Table S8).

**Figure 3:**
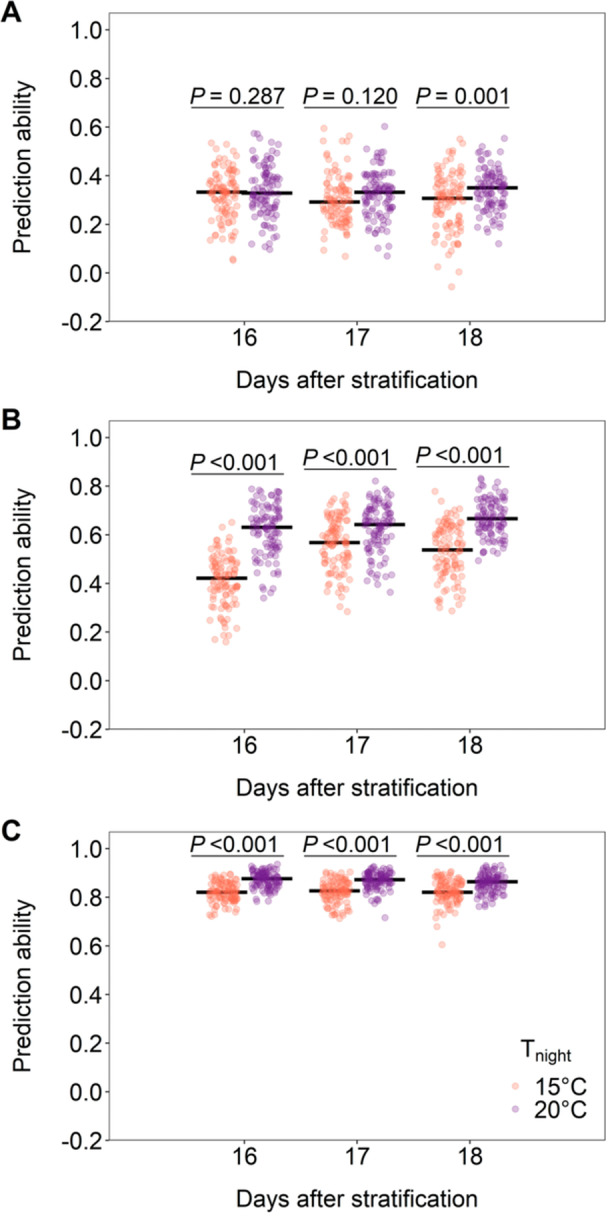
Prediction ability of F_q_’/F_m_’. The F_q_’/F_m_’ data of 293 accessions were used to train gBLUP models based on 211,771 SNPs (A), GWAS‐derived SNPs with ‐log_10_ (*P*) ≥ FDR threshold and/or ‐log_10_ (*P*) > 4.0 on at least two days (B), or with ‐log_10_ (*P*) ≥ 3.0 (C). Horizontal bars show the median of 100 cross validation replications. Circles represent individual replications in 15°C (coral) or 20°C (purple) T_night_. Statistically significant differences in prediction ability between the treatments were tested by two‐tailed *t*‐test. [Color figure can be viewed at wileyonlinelibrary.com]

Prediction ability, defined as the median Pearson correlation coefficient between the actual and the predicted F_q_’/F_m_’ across all cross validation runs, varied substantially depending on the SNP sets used (Figure 3). Low median prediction ability (~0.33) was obtained by using the 211,771 SNPs, with large variations across the test sets (Figure 3A). Since gBLUP assumes equal contributions of individual genetic markers to the phenotype, we tested whether the prediction can be improved by the Bayesian approach that allows for differential distribution of marker effects (Meuwissen et al. 2001). The results, however, indicated no improvement in predictive performance by Bayes A and Bayes B compared to gBLUP (Supporting Information S1: Table S9). Next, we used the information of the second set in the gBLUP model. This SNP set moderately increased the prediction ability, yielding a median prediction ability of 0.51 in 15°C T_night_ and 0.65 in 20°C T_night_, but the performance was still varying widely among the test sets (Figure 3B). Integrating the third set, which included a larger number of less strongly associated SNPs compared to the second set, markedly improved the prediction ability to reach above 0.8, while at the same time diminishing the disparity among the test sets in both conditions (Figure 3C).

The higher prediction ability by the third set than the first set is attributable to higher SNP‐trait associations; predictions based on the same number of randomly selected, weakly associated SNPs (‐log_10_ (*P*) < 3.0) showed poor prediction abilities compared to the SNPs in the third set (‐log_10_ (*P*) ≥ 3.0) (Supporting Information S1: Figure S5). The higher prediction ability by the third set than the second set, on the other hand, may be due to the tendency of gBLUP to predict the phenotype more accurately with increasing number of SNPs even if each SNP explains only a small proportion of the variance (Tan et al. 2017; Stich and Van Inghelandt 2018). Indeed, when the prediction ability was compared between the second and the third sets based on the same number of SNPs as in the second set (see Supporting Information S1: Table S8 for the number of SNPs), randomly chosen SNPs from the third set underperformed in 20°C T_night_ (Supporting Information S1: Figure S6B). It seems that the inclusion of additional SNPs in the third set, despite the moderate significance of their associations, increased the prediction ability in this condition. In contrast, the prediction ability in 15°C T_night_ was comparable between the second set and the same number of randomly chosen SNPs from the third set (Supporting Information S1: Figure S6A). This can be explained by the relatively modest ‐log₁₀ (*P*) values of most SNPs in the second set in 15°C Tₙᵢ_g_ₕₜ (≈4–5; Supporting Information S1: Table S7), which are not particularly higher than those of the third set (≥ 3.0). Inadequate correction for population structure can be ruled out to account for the high prediction ability with the third set, since no obvious clustering of accessions was observed in the PC analysis (Supporting Information S1: Figure S7) and neither PC1 nor PC2 was correlated with the predicted F_q_’/F_m_’.

Hence, the high prediction ability achieved by the third set of SNPs most likely reflects their collective influence on midday F_q_’/F_m_’ in the accessions under the respective T_night_ conditions.

### 3.4 SNPs Associated With Long‐Term Response of F_q_’/F_m_’ to T_night_

**Figure 4:**
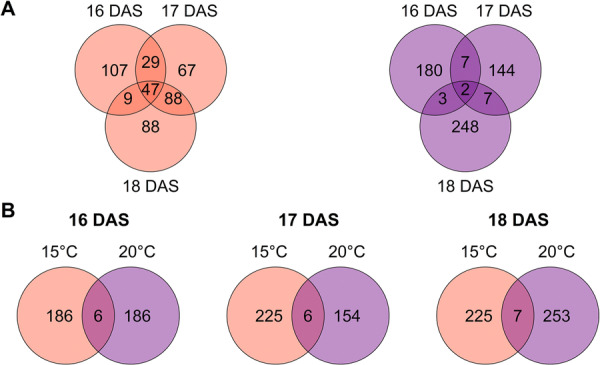
Shared and unique SNPs with ‐log_10_ (*P*) ≥ 3.0 in each T_night_ condition and measurement day. Overlapping SNPs between the three measurement days for each T_night_ condition (A) and between the two conditions on each day (B). Plants were growing in 15°C (coral) or 20°C (purple) T_night_. [Color figure can be viewed at wileyonlinelibrary.com]

While the third set of SNPs with ‐log_10_ (*P*) ≥ 3.0 gave equally high prediction abilities in both T_night_ conditions on all 3 days (Figure 3C), the composition of associated SNPs was changing substantially from day to day (Figure 4A). Nevertheless, part of the SNPs was repeatedly detected in 15°C T_night_, with larger overlaps found between two consecutive days (33%–58%) than between 16 and 18 DAS (29% and 24%). In total, 47 SNPs were associated with F_q_’/F_m_’ on all 3 days in 15°C T_night_. These 47 SNPs together explained nearly half of the F_q_’/F_m_’ variance in 15°C T_night_, each explaining 3% to 10% on different days (Table 1; Supporting Information S1: Table S10). The prediction based on the 47 SNPs was more accurate than using the first or the second set of SNPs, but not as good as the third set (Supporting Information S1: Table S11).

**Table 1:** 47 SNPs identified by GWAS with ‐log_10_ (*P*) ≥ 3.0 on three consecutive days in 15°C T_night_.

SNP	Allele 1/2	Col‐0 allele	MAF (%)	‐log_10_ (*P*)	Effect	*r* ^2^ (%)	Locus	Gene annotation
Chr1_846217	A/C	C	41.30	4.40	0.006	10.02	AT1G03410	2‐oxoglutarate and Fe(II)‐dependent oxygenase superfamily protein
Chr1_873447	A/T	A	34.47	4.04	−0.005	9.09	AT1G03495	HXXXD‐type acyl‐transferase family protein
Chr1_884146	G/A	G	37.54	3.88	−0.005	7.36	AT1G03540	pentatricopeptide repeat (PPR‐like) superfamily protein
Chr1_6478834	A/T	A	23.89	3.51	0.006	3.03	AT1G18790	RWP‐RK DOMAIN‐CONTAINING 1
Chr1_10027869	T/C	T	9.22	3.13	0.007	6.31	*	*
Chr1_19515673	C/G	C	37.88	3.49	0.005	7.55	AT1G52400	BETA GALACTOSIDASE 18
Chr1_23436110	G/C	G	6.83	3.40	−0.009	5.09	*	*
Chr2_1138854	C/T	C	12.29	4.11	−0.008	7.91	AT2G03730	ACT DOMAIN REPEAT 5 (ACR5)
(Chr2_1138985)	C/T	C	11.95	4.69	−0.008	8.91	AT2G03730	ACT DOMAIN REPEAT 5 (ACR5)
Chr2_1139010	A/C	A	14.68	3.65	−0.007	6.82	AT2G03730	ACT DOMAIN REPEAT 5 (ACR5)
Chr2_2884161	C/T	T	31.40	3.42	−0.005	3.97	AT2G06980	transposable element
Chr2_8145104	A/G	G	21.16	3.98	0.006	7.94	AT2G18800	XYLOGLUCAN ENDOTRANSGLUCOSYLASE/HYDROLASE 21
Chr2_11170979	A/G	A	2.05	3.76	−0.016	8.75	AT2G26250	3‐KETOACYL‐COA‐SYNTHASE 10
Chr2_13684149	G/A	G	13.65	3.48	−0.006	6.62	AT2G32235	protein coding
Chr2_14059450	C/A	C	29.01	3.10	−0.005	6.32	AT2G33170	leucine‐rich repeat receptor‐like protein kinase family protein
Chr3_1275424	C/T	C	16.72	3.83	0.007	3.97	AT3G04690	malectin/receptor‐like protein kinase family protein
Chr3_3395109	C/G	C	9.56	3.36	0.008	7.84	AT3G10845	RNA‐binding family protein
Chr3_6651631	A/C	A	21.16	3.08	−0.005	5.86	*	*
Chr3_9636722	T/G	T	34.81	3.62	−0.005	7.19	*	*
Chr3_10582418	C/T	T	47.44	3.62	0.005	2.65	AT3G28320	UPF0496 protein
Chr3_10874763	T/A	A	39.93	3.09	−0.004	3.32	AT3G28860	ATP BINDING CASETTE B19
Chr3_10898637	G/A	G	28.67	3.39	−0.005	7.79	AT3G28890	RECEPTOR LIKE PROTEIN 43
Chr3_16153315	T/C	T	45.73	3.97	0.005	6.60	AT3G44560	FATTY ACID REDUCTASE 8
Chr3_19054365	A/G	A	3.07	3.76	−0.014	8.16	AT3G51330	eukaryotic aspartyl protease family protein
Chr3_19257874	T/C	T	9.56	3.05	0.007	5.40	*	*
Chr3_21427556	G/A	G	12.63	3.32	−0.007	6.63	AT3G57860	OMISSION OF SECOND DIVISION
Chr3_21432797	C/T	C	21.16	3.70	−0.006	6.99	AT3G57880	MULTIPLE C2 DOMAIN AND TRANSMEMBRANE REGION PROTEIN 3 (MCTP3)
(Chr3_21432851)	C/T	C	21.84	3.58	−0.006	6.97	AT3G57880	MULTIPLE C2 DOMAIN AND TRANSMEMBRANE REGION PROTEIN 3 (MCTP3)
Chr3_22377074	A/G	A	36.86	3.46	−0.005	5.51	*	*
Chr4_5411416	C/G	C	19.11	3.41	−0.006	6.78	AT4G08510	protein coding
Chr4_5633114	C/A	C	3.07	3.50	−0.013	7.46	AT4G08840	PUMILIO 11
Chr4_7788139	C/T	C	12.97	5.02	−0.008	9.56	AT4G13400	2‐oxoglutarate and Fe(II)‐dependent oxygenase superfamily protein
Chr4_14017514	C/T	C	15.36	3.29	−0.006	6.50	AT4G28310	microtubule‐associated protein
Chr4_14030315	T/G	T	16.38	3.08	−0.006	5.59	AT4G28360	RIBOSOMAL PROTEIN UL22MY
Chr4_15903731	C/G	C	2.05	3.36	−0.015	8.98	*	*
Chr4_18442418	A/G	A	12.63	3.92	−0.007	7.28	AT4G39756	galactose oxidase/kelch repeat superfamily protein
Chr5_700043	A/G	A	17.06	3.37	−0.006	3.73	*	*
Chr5_3284989	C/A	A	40.61	3.76	0.005	4.69	AT5G10450	14‐3‐3LAMBDA
Chr5_5536626	A/C	A	19.11	3.17	−0.006	7.95	AT5G16840	BINDING PARTNER OF ACID11 1
(Chr5_5538353)	G/A	G	18.09	3.16	−0.006	8.02	AT5G16850	TELOMERASE REVERSE TRANSCRIPTASE
Chr5_5620806	C/T	T	46.76	3.98	−0.005	6.94	AT5G17080	cysteine proteinases superfamily protein
Chr5_5627597	T/G	T	30.03	3.63	−0.005	6.27	*	*
Chr5_5627893	A/G	G	35.84	3.25	−0.004	4.64	*	*
Chr5_9760768	C/A	C	6.14	3.65	−0.010	6.71	AT5G27607	JAB1/Mov34/MPN/PAD‐1 ubiquitin protease
Chr5_18289242	A/G	G	24.57	3.81	−0.005	6.92	AT5G45200	disease resistance protein (TIR‐NBS‐LRR class) family
Chr5_21767983	T/C	T	3.07	3.14	−0.012	6.96	*	*
Chr5_23908100	C/A	A	46.42	3.31	0.004	6.31	AT5G59260	l‐TYPE LECTIN RECEPTOR KINASE II.1
Simultaneous fit						46.16		

*Note:* Shown are the associations of 47 SNPs with F_q_’/F_m_’ on 18 DAS. For the associations on 16 and 17 DAS, see Supporting Information S1: Table S10. Allele 1/2 shows the major/minor allele. The allele effects were estimated for the minor alleles and given in the unit of F_q_’/F_m_’. The *r* ^2^ values give the proportion of the variance explained by individual SNPs or a set of SNPs (simultaneous fit). For pairs of SNPs that were in linkage disequilibrium (underlined), only one of them (in parentheses) was included in the simultaneous fit. DAS, days after stratification; MAF, minor allele frequency.

* SNPs are located in intergenic regions.

The 47 SNPs in 15°C T_night_ are likely to be identified by GWAS combining F_q_’/F_m_’ data of all 3 days. To verify this, adjusted entry means of F_q_’/F_m_’ were computed for combined F_q_’/F_m_’ data, including measurement day as a random effect (Supporting Information S1: Figure S8A). The heritability of these adjusted entry means was 0.93, which is higher than the heritability calculated for each day separately (cf. Figure 1E). The GWAS based on the combined data (Supporting Information S1: Figure S8) confirmed the associations of 46 SNPs out of 47 (Supporting Information S1: Figure S9). Further, 159 SNPs, which were associated with F_q_’/F_m_’ on one or 2 days in the separate analysis, were also detected by the combined analysis. In addition, 15 unique SNPs had ‐log_10_ (*P*) ≥ 3.0 in the combined analysis. A single SNP located in AT3G05050, encoding a protein kinase superfamily protein, passed the FDR threshold of 0.05 in the GWAS across 3 days (Supporting Information S1: Figure S8B). This SNP was identified on 17 and 18 DAS in the separate analysis (SNP No. 7 in Figure 2).

We consider the 47 SNPs in Table 1 as the core SNPs associated with F_q_’/F_m_’ variation among the Arabidopsis accessions in the 15°C T_night_ condition. None of these SNPs was found in loci known to be directly involved in photosynthesis. Besides the SNP in 3‐KETOACYL‐COA SYNTHASE 10 having the largest modulus effect of −0.016, five SNPs had the effect size between −0.01 and −0.015 (Table 1). Two of them are located in AT3G51330 and AT5G27607 which encode a eukaryotic aspartyl protease family protein and a JAB1/Mov34/MPN/PAD‐1 ubiquitin protease, respectively. Another SNP was found in AT4G08840 coding for PUMILIO 11, a member of sequence‐specific RNA‐binding PUF proteins which regulate mRNA stability and translation (Joshna et al. 2020). The other two SNPs are in intergenic regions. Notably, two genes had multiple associated SNPs identified across all 3 days (Table 1). ACT DOMAIN REPEAT 5 (ACR5; AT2G03730) had three associated SNPs, one of which was also significant in 20°C T_night_ (SNP No. 2 in Figure 2), and MULTIPLE C2 DOMAIN AND TRANSMEMBRANE REGION PROTEIN 3 (MCTP3; AT3G57880) had two SNPs.

In striking contrast to 15°C T_night_, between‐day overlaps of SNP‐trait associations were no more than 2%–6% in 20°C T_night_, as little as the overlaps between the two T_night_ conditions on each day (3%–4%) (Figure 4). This pronounced day‐to‐day variation in 20°C T_night_ may signify dynamic nature of SNP‐trait associations in this warm night condition, with distinct sets of SNPs affecting midday F_q_’/F_m_’ on each day. Another possible explanation could be day‐to‐day fluctuations of ‐log_10_ (*P*) values around the chosen threshold, resulting in inclusion or exclusion of SNPs. If the former is the case, the SNPs identified on 16 or 17 DAS, for instance, will not allow accurate prediction of F_q_’/F_m_’ on 18 DAS (see the poor prediction ability of randomly chosen SNPs with ‐log_10_ (*P*) < 3.0 in Supporting Information S1: Figure S5). In the latter case, on the other hand, we may find reasonably good predictions. Judging by the Pearson correlation coefficients between the actual and the predicted F_q_’/F_m_’, predictive performance for 18 DAS in 20°C T_night_ declined when the SNPs from 16 or 17 DAS in the same T_night_ condition or 18 DAS in 15°C T_night_ were used as predictors (Supporting Information S1: Table S12). Nonetheless, these correlation coefficients were substantially higher than using the first and the second sets of SNPs from the corresponding day and T_night_ (Supporting Information S1: Table S11). The SNPs from non‐matching days in the same 20°C T_night_ gave better predictions than the SNPs from the same day but in 15°C T_night_. Also, the SNPs from 17 DAS did better than those from 16 DAS for predicting F_q_’/F_m_’ on 18 DAS in 20°C T_night_ (Supporting Information S1: Table S12). Because F_q_’/F_m_’ levels were similar between the plants grown in 15°C T_night_ on 18 DAS and those grown in 20°C T_night_ on 16 DAS (Figure 1A), we also compared the associated SNPs between these datasets (Supporting Information S1: Figure S10). The overlap was minimal and comparable to those observed between the two T_night_ conditions on the same day (Figure 4B), suggesting that a developmental shift is unlikely to explain the dissimilarity in associated SNPs between 15°C T_night_ and 20°C T_night_.

Taken together, these results imply that a greater number of loci than identified by ‐log_10_ (*P*) ≥ 3.0 on each day (Supporting Information S1: Table S8) were contributing to the F_q_’/F_m_’ variance among the Arabidopsis accessions, and that the associated loci were changing across days and T_night_ conditions. Between 15°C and 20°C T_night_, cooler nights led to a steady influence of a subset of associated SNPs on F_q_’/F_m_’, while no core set of SNPs was found in warmer nights.

### 3.5 gBLUP Outperforms Ensemble Methods in Predicting F_q_’/F_m_’

The GPs described above were all realised by gBLUP. Given the large number of associated SNPs and their potential interactions, non‐linear models may enable more accurate predictions than gBLUP. We therefore compared predictive performance of gBLUP with ensemble machine learning methods, namely, random forest (Ho 1995; Breiman 2001) and XGBoost (Chen et al. 2016). Both methods construct multiple individual models using different randomly sampled subsets of data. However, while random forest fits these models independently and in parallel, XGBoost fits them sequentially and iteratively, with each model correcting the errors of its predecessor (Chen et al. 2016). We leveraged the optimised framework of XGBoost to handle the 211,771 SNPs for predicting F_q_’/F_m_’. Random forest was used for prediction by the GWAS‐derived SNPs (i.e., the second and the third SNP sets). All models were trained by identical training sets and evaluated on the same validation sets to ensure comparability (Supporting Information S1: Figure S11).

The ensemble methods performed similarly to gBLUP in predicting F_q_’/F_m_’ by the 211,771 SNPs and the second set of SNPs (Table 2). The prediction of XGBoost is unlikely to be affected by population structure because adjusting the genotypes for population structure did not improve the prediction (Supporting Information S1: Table S13). As seen by gBLUP, the information on the GWAS‐derived SNPs improved predictive performance of the ensemble methods in both 15°C and 20°C T_night_ (Table 2). The best prediction was achieved by the third set of SNPs with ‐log_10_ (*P*) ≥ 3.0 in both T_night_ on all 3 days, whereby gBLUP outperformed random forest. Specifically, gBLUP produced higher correlations between the actual and predicted F_q_’/F_m_’, whereas random forest tended to overestimate in accessions with low F_q_’/F_m_’ and slightly underestimate in those with high F_q_’/F_m_’, yielding flatter regression slopes (Supporting Information S1: Figure S12).

**Table 2:** Performance of gBLUP and ensemble methods in predicting F_q_’/F_m_’.

T_night_	Method	DAS	Predictors					
			211,771 SNPs		FDR threshold		‐log_10_ (*P*) ≥ 3.0	
			RMSE	COR	RMSE	COR	RMSE	COR
15°C	gBLUP	16	0.022 ± 0.001	0.20 ± 0.13	0.021 ± 0.001	0.32 ± 0.06	0.014 ± 0.001	0.79 ± 0.01
		17	0.022 ± 0.002	0.23 ± 0.06	0.020 ± 0.001	0.48 ± 0.08	0.012 ± 0.000	0.84 ± 0.04
		18	0.021 ± 0.002	0.23 ± 0.02	0.018 ± 0.000	0.51 ± 0.12	0.012 ± 0.001	0.82 ± 0.06
	random forest or XGBoost	16	0.023 ± 0.002	0.09 ± 0.06	0.022 ± 0.001	0.29 ± 0.03	0.018 ± 0.002	0.63 ± 0.08
		17	0.023 ± 0.003	0.04 ± 0.05	0.021 ± 0.002	0.39 ± 0.07	0.018 ± 0.002	0.71 ± 0.05
		18	0.021 ± 0.002	0.16 ± 0.05	0.018 ± 0.001	0.48 ± 0.12	0.016 ± 0.001	0.74 ± 0.07
20°C	gBLUP	16	0.022 ± 0.001	0.15 ± 0.09	0.016 ± 0.001	0.69 ± 0.07	0.010 ± 0.001	0.89 ± 0.02
		17	0.018 ± 0.001	0.25 ± 0.01	0.015 ± 0.001	0.62 ± 0.01	0.009 ± 0.000	0.86 ± 0.03
		18	0.016 ± 0.001	0.21 ± 0.03	0.013 ± 0.001	0.59 ± 0.08	0.008 ± 0.001	0.86 ± 0.03
	random forest or XGBoost	16	0.022 ± 0.001	0.22 ± 0.10	0.018 ± 0.001	0.60 ± 0.07	0.017 ± 0.001	0.71 ± 0.02
		17	0.017 ± 0.002	0.27 ± 0.06	0.015 ± 0.001	0.54 ± 0.06	0.013 ± 0.001	0.72 ± 0.04
		18	0.016 ± 0.001	0.30 ± 0.04	0.014 ± 0.001	0.51 ± 0.09	0.013 ± 0.001	0.65 ± 0.02

*Note:* Predictors were 211,771 SNPs, GWAS‐derived SNPs with ‐log_10_ (*P*) ≥ FDR threshold (0.05) and/or ‐log_10_ (*P*) > 4.0 on at least two days, or with ‐log_10_ (*P*) ≥ 3.0. Prediction of the ensemble methods was performed with random forest when based on the GWAS‐derived SNPs and with XGBoost when based on the 211,771 SNPs. Validation of the XGBoost model in the testing set was performed using parameters selected in a grid search (see Supporting Information S1: Table S3). The different models were trained on the same training sets and evaluated in the same test sets. Performance was assessed using root mean squared error (RMSE) and Pearson correlation coefficient (COR) averaged across three testing sets (± SE). DAS, days after stratification; FDR, false discovery rate; T_night_, night temperature.

### 3.6 Validation Experiment Confirmed F_q_’/F_m_’ Predictions in Independent and Genetically Diverse Accessions

**Figure 5:**
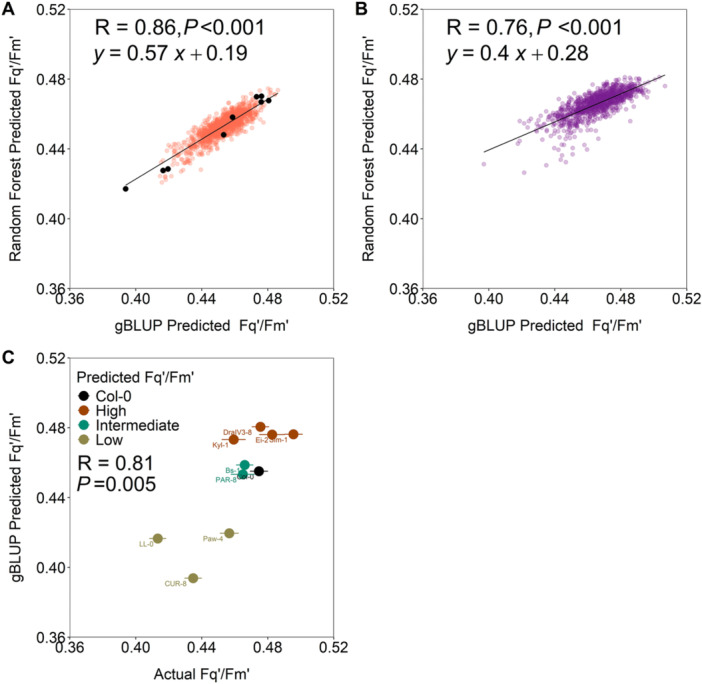
Genomic prediction of F_q_’/F_m_’ and experimental validation. Prediction in 1,014 non‐phenotyped accessions using gBLUP or random forest based on the GWAS‐derived SNPs with ‐log_10_ (*P*) ≥ 3.0 in 15°C (A) or 20°C (B) T_night_ on 18 days after stratification (DAS). Circles represent predicted F_q_’/F_m_’ of individual accessions. For nine accessions (marked with black circles in panel A; listed in Supporting Information S1: Table S2), prediction by gBLUP was experimentally validated in the 15°C T_night_ condition (C). Columbia‐0 (Col‐0) was included as reference in the validation experiment. Different colours of the accessions in panel C denote low, intermediate, and high predicted F_q_’/F_m_’ levels. Horizontal error bars are ±SE of the adjusted entry means of actual F_q_’/F_m_’ of individual accessions. R indicates the Pearson correlation coefficient and *P* the significance. The number of replicate plants per accession was: Bs‐1, *n* = 14; CUR‐8, *n* = 19; DralIV3‐8, *n* = 18; Ei‐2, *n* = 3; Kyl‐1, *n* = 4; LL‐0, *n* = 25; PAR‐8, *n* = 4; Paw‐4, *n* = 11; Sim‐1, *n* = 11; Col‐0, *n* = 14. For validation on 16 and 17 DAS, see Supporting Information S1: Figure S14. [Color figure can be viewed at wileyonlinelibrary.com]

Encouraged by the high accuracy of prediction using the GWAS‐derived SNPs with ‐log_10_ (*P*) ≥ 3.0 (Figure 3C; Table 2), we proceeded to examine whether the models, which were trained and tested on the 293 accessions, can predict F_q_’/F_m_’ for genetically diverse Arabidopsis accessions in 15°C and 20°C T_night_. Thus, the above‐described gBLUP and random forest models with the third SNP set were applied to 1,014 accessions from the RegMap population (Horton et al. 2012). The F_q_’/F_m_’ values predicted by gBLUP showed similar distributions in the 293 accessions and the 1,014 accessions under both T_night_ conditions (Supporting Information S1: Figure S13). The predictions by the linear and non‐linear methods were largely comparable for the 1,014 accessions, although random forest predicted higher F_q_’/F_m_’ values than gBLUP for accessions with low F_q_’/F_m_’ (Figure 5A,B), as was seen during the cross validation in the 293 accessions (Supporting Information S1: Figure S12).

We then conducted an experiment to validate the F_q_’/F_m_’ predictions. The 15°C T_night_ condition was chosen for the validation experiment since the correlation between gBLUP and random forest predictions was higher and the difference between the highest and the lowest predicted F_q_’/F_m_’ was larger in 15°C than in 20°C T_night_ (Figure 5A,B), although heritability of F_q_’/F_m_’ was lower (Figure 1E) and model predictions were less accurate in 15°C T_night_ (Table 2). Nine accessions having low, intermediate and high predicted F_q_’/F_m_’ (Figure 5A; Supporting Information S1: Table S2) were grown alongside the reference genotype Col‐0 and midday F_q_’/F_m_’ was measured on 16, 17 and 18 DAS. Overall, the predicted F_q_’/F_m_’ values were in very good agreement with the values observed in the accessions (R = 0.81 and *p* = 0.005 in Figure 5C; Supporting Information S1: Figure S14). While marginal differences (ΔF_q_’/F_m_’ < 0.03) between high and intermediate accessions were difficult to verify experimentally, two accessions having the lowest F_q_’/F_m_’ (LL−0 and CUR‐8) were correctly identified on all 3 days, supporting the models’ robustness and applicability across diverse populations.

## 4 Discussion

Temperature profoundly influences biological processes. Up to an optimal point, rising temperatures accelerate biochemical and physiological reactions, whereas lower temperatures slow down enzyme activity and cellular processes, limiting growth and development. Reduced sink activity (i.e., the activity of a non‐photosynthesising organ or tissue to use or store photoassimilates) and the resulting sugar accumulation in leaves can trigger feedback suppression of photosynthesis (Paul and Foyer 2001; Körner 2015). In accordance, Arabidopsis plants grown in 15°C T_night_ exhibited smaller rosettes and lower midday F_q_’/F_m_’ than those in 20°C T_night_ (Figure 1A,C). Since F_q_’/F_m_’ was measured under identical conditions for both T_night_ treatments, lower F_q_’/F_m_’ observed in 15°C T_night_ suggests feedback regulation of photosynthesis by nocturnal sink limitation. Similar reductions in photosynthetic efficiency under low T_night_ have been reported in grapevine (Tombesi et al. 2019), sugar beet (Wang et al. 2022), and cotton (Warner et al. 1995). Low T_night_ may also delay the development of photosynthetic capacity; the distribution of F_q_’/F_m_’ across accessions was comparable between 18 DAS in 15°C T_night_ and 16 DAS in 20°C T_night_ (Figure 1A; Supporting Information S1: Table S5), although the associated SNPs were not similar at all between these datasets (Supporting Information S1: Figure S10).

The HapMap accessions (Li et al. 2010) used in this study are exposed to a broad range of minimum temperatures in their native environments. Based on the estimates by Lasky et al. (2012), mean monthly minimum temperatures during the predicted growing season range from −0.2°C to 11.32°C for the majority of the accessions used in this study (231 out of 308). Accordingly, 15°C T_night_ is closer to their native growth environments, whereas 20°C T_night_ represents a very warm nocturnal temperature. Despite this large departure from the native conditions, Arabidopsis plants generally exhibited increased growth under 20°C T_night_ (Figure 1C) accompanied by higher F_q_’/F_m_’ (Figure 1A), indicating a positive effect of warmer nights during the vegetative growth stage. Nevertheless, predawn F_v_/F_m_ was slightly lower in the plants grown in 20°C than 15°C T_night_ (Figure 1B), possibly reflecting a more reduced plastoquinone pool in the thylakoid membrane by reducing agents from mitochondria and/or stromal metabolism during warmer nights (Hoefnagel et al. 1998; Scheibe 2019).

While both growth and PSII efficiency were influenced by T_night_ and genetic effects, F_q_’/F_m_’ stood out with the highest heritability of all parameters measured in this study (Figure 1E). Broad‐sense heritability represents the proportion of total phenotypic variance attributed to genetic variance, with higher values indicating stronger genetic control. In fact, the genetic variance component of F_q_’/F_m_’ was statistically significant (*p* < 0.001) on all 3 days in both 15°C and 20°C T_night_. Interestingly, heritability increased from 16 to 18 DAS for all parameters except RGR in 15°C T_night_ (Figure 1E). Temporal changes in heritability–throughout the day, across developmental stages, and over growing seasons–have been reported for photosynthesis‐ and growth‐related traits in Arabidopsis (Flood et al. 2016), canola (Knoch et al. 2020), and barley (Gao et al. 2024). The lower heritability observed on 16 DAS is consistent with greater environmental sensitivity and higher ontogenetic variation in younger plants (Henn and Damschen 2021), which often reduces heritability (Tonsor et al. 2013). Another intriguing observation is the opposite effects of T_night_ on heritability of PSII efficiency and growth; F_q_’/F_m_’ and F_v_/F_m_ showed lower heritability in 15°C T_night_ whereas the heritability of projected rosette area and especially RGR was higher in this condition (Figure 1E). Lower heritability of PSII efficiency in 15°C T_night_ may be due to slower development and/or higher environmental sensitivity of photosynthesis in this condition. Higher heritability of growth in 15°C T_night_, on the other hand, is difficult to explain by development. Cooler nights, albeit closer to the native growth conditions, may create a more selective environment for growth, amplifying genetic differences among accessions that are less pronounced in warmer T_night_.

Our GWAS revealed another distinct effect of cooler nights on the genetic control of F_q_’/F_m_’, manifested as the steady contributions of a subset of associated SNPs (Table 1, Supporting Information S1: Table S10). None of the 47 core SNPs identified in 15°C T_night_ is located in loci that are known to be directly involved in photosynthesis, suggesting a broad influence of multifaceted components and reactions on F_q_’/F_m_’. This finding is in line with the previous GWAS study in Arabidopsis, reporting the absence of photosynthetic genes among 63 candidates that were associated with high‐light response of F_q_’/F_m_’ (called Φ_PSII_; van Rooijen et al. 2015). Of the 47 core SNPs, three are located in ACR5 and two in MCTP3 (Table 1). ACR5 belongs to a small family of genes encoding ACT domain repeat proteins (Hsieh and Goodman 2002). The ACT domain is a regulatory module that binds small molecules—mostly amino acids—and functions in controlling metabolism, transport, and signal transduction (Grant 2006). High levels of ACR5 mRNA have been detected in stems of 6‐week‐old Arabidopsis plants, and a 48‐h dark treatment increased the mRNA levels in 2‐week‐old plants (Hsieh and Goodman 2002). Given its co‐expression with several Type‐A Arabidopsis RESPONSE REGULATOR genes (ARR3, 4, 6, 7, and 9) (ATTED‐II version 13.0; https://atted.jp), ACR5 may play a role in cytokinin‐dependent regulation of growth and development. Members of MCTP family proteins localise to plasmodesmata (Vaddepalli et al. 2014; Brault et al. 2019). Proteins of MCTP3 (aka FT INTERACTING PROTEIN 3, FTIP3), MCTP4 (FTIP4), and MCTP6 act as tethers between endoplasmic reticulum and plasma membrane specifically at plasmodesmata (Brault et al. 2019), presumably stabilising nascent plasmodesmata during cytokinesis (Li et al. 2024). Higher‐order loss‐of‐function mutants of these genes (*mctp3mctp4* and *mctp3mctp4mctp6*) exhibit reduced plasmodesma density compared with wild type (Li et al. 2024). The possible influence of ACR5 and MCTP3 on F_q_’/F_m_’ remains to be investigated in future studies.

Contrary to the picture in 15°C T_night_, only a few SNPs were repeatedly identified on different days in 20°C T_night_ (Figure 4A). Since gBLUP showed fairly good predictive performance using SNPs from non‐matching days for this T_night_ condition (Supporting Information S1: Table S12), much better than using all 211,771 SNPs (Table 2; Supporting Information S1: Table S11) and randomly selected less significant SNPs (Supporting Information S1: Figure S5), it is unlikely that 20°C T_night_ caused radical day‐to‐day shifts in the genetic architecture of F_q_’/F_m_’. Rather, it seems to reflect the existence of numerous small‐effect loci, far beyond the 160‐260 SNPs identified by ‐log_10_ (*P*) ≥ 3.0 on each day (Supporting Information S1: Table S8). The higher predictive performance obtained by the SNPs from the same T_night_ than the other T_night_, and from the previous day than 2 days earlier (Supporting Information S1: Table S12), underscores the roles of T_night_ and development in shaping the genetic basis of F_q_’/F_m_’

The multitude of small‐effect variants associated with F_q_’/F_m_’ aligns with the omnigenic model (Boyle et al. 2017) which posits that any gene expressed in relevant cells can indirectly influence a complex trait by modulating the expression or activity of primary genes having direct effects. Photosynthesis may exemplify this principle; it relies on many enzymes and cofactors whose synthesis, regulation, and degradation depend on an extensive network of other enzymes, transcription factors, and transporters, themselves subject to similar control. Close interconnections between photosynthesis, central metabolism, and growth further strengthen the omnigenic‐like nature of photosynthesis. The very fact that midday F_q_’/F_m_’ and its genetic architecture changed in response to growth T_night_, presumably via changes in nocturnal sink activities (sink regulation), highlights the inherent complexity of this trait. Distributing control over many enzymes allows metabolic flux, like in photosynthesis and related pathways, to be regulated efficiently with minimal disruption and perturbation (Fell and Thomas 1995). This system‐level control arises from synergistic interactions among multiple control sites–a concept that may also apply to the genetic control of complex traits where numerous small‐effect loci collectively influence phenotypic outcomes under changing environmental conditions and at different developmental stages.

When a trait is highly polygenic, how can SNP‐trait associations be meaningfully evaluated? Assessing a limited number of loci by mutant characterisation seems insufficient, since individual SNPs exert only marginal effects and collective influence of the majority is neglected. We therefore adopted a holistic approach that focuses on the combined contribution of many SNPs, considering SNP‐trait associations to be reliable if they yield significant improvements in the performance of GP models. The results demonstrated that the GWAS‐derived SNPs, particularly those selected by ‐log_10_ (*P*) ≥ 3.0, substantially and consistently enhanced the prediction of F_q_’/F_m_’ in the 293 accessions (Figure 3C) compared to using all (Figure 3A; Table 2; Supporting Information S1: Table S11) or random SNPs (Supporting Information S1: Figure S5). The prediction was improved regardless of whether linear or non‐linear models were applied, although gBLUP outperformed random forest (Table 2; Supporting Information S1: Figure S12). Increases in GP accuracy following the incorporation of GWAS results have been reported in different plant species such as rice (Spindel et al. 2016), maize (Bian and Holland 2017), wheat (Sehgal et al. 2020), and poplar (Guo et al. 2025). Without GWAS‐derived information (i.e., using all 211,771 SNPs), the median prediction ability of gBLUP for F_q_’/F_m_’ was ~0.33 (Figure 3A), similar to the previous study in which gBLUP achieved the accuracy of ~0.3 and ~0.4 for predicting Φ_PSII_ (F_q_’/F_m_’) in 344 Arabidopsis accessions under light intensities of 100 and 550 μmol photons m^−2^ s^−1^, respectively (Farooq et al. 2021). Neither Bayes A nor Bayes B improved the predictive performance (Supporting Information S1: Table S9), despite their capacity to model heterogenous marker effects (Meuwissen et al. 2001). This agrees with previous findings that gBLUP and Bayes methods performed similarly when traits were governed by numerous small‐effect loci (Daetwyler et al. 2013; Meher et al. 2022). The limited accuracy achieved by genome‐wide SNPs could be due to the introduction of noise by the vast majority of non‐associated SNPs. Conversely, small sets of SNPs identified by stringent thresholds (‐log_10_ (*P*) ≥ FDR and/or ‐log_10_ (*P*) > 4.0 on at least 2 days) yielded rather modest and inconsistent gains in prediction ability (Figure 3B; Table 2; Supporting Information S1: Table S11), suggesting that these SNPs alone cannot sufficiently account for the observed variation in F_q_’/F_m_’ across the accessions. Hence, conventional reliance on conservative thresholds may not be good practice for GWAS on complex traits underpinned by numerous small‐effect loci when aiming to maximise the prediction ability. A more effective strategy could be to systematically assess multiple significance thresholds and identify the sets of associated markers that allow high prediction accuracy.

Whilst predictions can be significantly improved by incorporating GWAS‐derived information, the chief purpose of GP is to predict the potential of individuals based on their genome‐wide marker data alone. Yet, GP models may not transfer well across genetically diverse populations, as prediction ability is strongly influenced by kinship, population structure, and the extent of linkage disequilibrium (Alemu et al. 2024). Thus, to evaluate cross‐population applicability, we tested the GWAS‐based models, which were trained on the 293 phenotyped accessions from the HapMap population (Li et al. 2010), against 1,014 non‐phenotyped accessions from the RegMap population (Horton et al. 2012). Using the SNPs with ‐log_10_ (*P*) ≥ 3.0 as predictors, the predictions of gBLUP and random forest were strongly and linearly correlated in the 1,014 accessions under both 15°C and 20°C T_night_ (Figure 5A,B). To further assess model performance, we experimentally validated the predictions in a subset of accessions spanning a range of predicted F_q_’/F_m_’ values (Figure 5A; Supporting Information S1: Table S2). Albeit rarely performed in GP studies, experimental validation offers a definitive assessment of model performance in a given context. Our results confirmed the GP models' capacity to correctly identify genotypes with low F_q_’/F_m_’ (LL−0 and CUR‐8) in the 15°C T_night_ condition, whereas minimal differences between high and intermediate genotypes could not be verified by the measurements (Figure 5C; Supporting Information S1: Figure S14). Accordingly, the GWAS‐derived SNPs seem to capture sufficient genetic information to reliably identify low‐F_q_’/F_m_’ individuals in an independent, genetically diverse Arabidopsis population under our growth conditions. Moreover, we noticed that a previous study by Meyer et al. (2023), which had been conducted under conditions distinct from ours, also reported low Φ_PSII_ (F_q_’/F_m_’) in LL−0, suggesting a certain degree of transferability of the models even across environments.

In summary, our findings uncover the highly polygenic architecture of photosynthesis and its differential responses to warmer versus cooler nights. By directly influencing nocturnal sink activities such as growth and respiration, T_night_ reveals that sink regulation of photosynthesis extends beyond the phenotype to the genetic level. The results provide a framework for leveraging GWAS and GP to explore complex quantitative traits, such as photosynthesis, toward breeding crops that are resilient to future climate challenges.

## Conflicts of Interest

The authors declare no conflicts of interest.

## Supporting information

Supporting File 1

**Figure S1:** Scheme of plant positions in the inlets.
**Figure S2:** Population structure of the 293 Arabidopsis accessions.
**Figure S3:** Q‐Q plots of the observed and the expected p values distribution from the GWAS on Fq’/Fm’ in the 293 accessions on 16, 17 and 18 days after stratification.
**Figure S4:** Manhattan plots of the GWAS on Fq’/Fm’ in the 293 accessions.
**Figure S5:** Distribution of prediction ability of gBLUP models for Fq’/Fm’ based on the GWAS‐derived SNPs with −log_10_ (P) < 3.0 or ≥ 3.0.
**Figure S6:** Distribution of prediction ability of gBLUP models for Fq’/Fm’ based on random GWAS‐derived SNPs with −log_10_ (P) ≥ 3.0 and SNPs with −log1_0_ (P) ≥ FDR threshold (0.05) and/or −log_10_ (P) > 4.0 on at least two days.
**Figure S7:** Population structure of the 293 accessions color‐coded according to the Fq’/Fm’ predicted by gBLUP.
**Figure S8:** Analysis of Fq’/Fm’ and its associated SNPs in 293 accessions in 15°C T_night_ across the three measurement days combined.
**Figure S9:** Shared and unique SNPs with −log_10_ (P) ≥ 3.0 between the three measurement days analyzed separately and across the three days combined in the 15°C T_night_ condition.
**Figure S10:** Shared and unique SNPs with −log_10_ (P) ≥ 3.0 between the plants growing in 15°C T_night_ on 18 days after stratification (DAS) and those growing in 20°C T_night_ on 16 DAS.
**Figure S11:** Population structure of randomly selected accessions for training and testing gBLUP models of Fq’/Fm’.
**Figure S12:** Performance of gBLUP and random forest in predicting Fq’/Fm’ on 18 days after stratification (DAS).
**Figure S13:** Distribution of predicted Fq’/Fm’ in the 293 phenotyped accessions in the GWAS panel and 1,014 non‐phenotyped accessions from the RegMap panel.
**Figure S14:** Experimental validation of the prediction of Fq’/Fm’ by gBLUP in the 15°C T_night_ condition.
**Table S1:** List of 308 Arabidopsis thaliana accessions.
**Table S2:** List of nine accessions in the validation experiment.
**Table S3:** Hyperparameters evaluated in the grid search to maximize the predictive performance of XGBoost.
**Table S4:** Variability in the effective (Fq’/Fm’) and the maximal efficiency (Fv/Fm) of photosystem II, projected rosette area, and relative growth rate (RGR) among replicates of Col‐0.
**Table S5:** Descriptive statistics of the adjusted entry means of the effective (Fq’/Fm’) and the maximal efficiency (Fv/Fm) of photosystem II, projected rosette area, and relative growth rate (RGR).
**Table S6:** Variability in the adjusted entry means and model residuals among plant replicates for the effective (Fq’/Fm’) and the maximal efficiency (Fv/Fm) of photosystem II, projected rosette area, and relative growth rate (RGR).
**Table S7:** Single nucleotide polymorphisms (SNPs) associated with long‐term response of Fq’/Fm’ to night temperature.
**Table S8:** Number of single nucleotide polymorphisms (SNPs) used as predictors of Fq’/Fm’.
**Table S9:** Performance of gBLUP and Bayesian approaches in predicting Fq’/Fm’ for the 293 Arabidopsis accessions in the GWAS panel.
**Table S10:** 47 SNPs identified by GWAS with −log_10_ (P) ≥ 3.0 on three consecutive days in 15°C T_night_.
**Table S11:** Performance of gBLUP in predicting Fq’/Fm’ for the Arabidopsis GWAS panel in 15°C T_night_ based on four different sets of SNPs.
**Table S12:** Performance of gBLUP in predicting Fq’/Fm’ for the Arabidopsis GWAS panel in 20°C T_night_ on 18 days after stratification (DAS) based on SNPs identified in the same T_night_ but on non‐matching days, or on the same day but in 15°C T_night_.
**Table S13:** Performance of XGBoost in predicting Fq’/Fm’ based on the genotype data adjusted and non‐adjusted for population structure.

## Data Availability

The data that support the findings of this study are openly available in Data Publication Server of Forschungszentrum Jülich at https://datapub.fz-juelich.de/plantsciences/Genomic_Prediction_Arabidopsis/.
