# Supplementary material for: Highly Polygenic Control of Photosynthetic Responses to Nighttime Temperature Studied by Genomic Prediction
Source: Plant Cell Environ. 2026 May 5;49(8):5886–903. doi: 10.1111/pce.70577 (PMC13353682; doi:10.1111/pce.70577)
Supplement: Supplementary file 1 — Supporting File 1 [file PCE-49-5886-s001.pdf]

Highly polygenic control of photosynthetic responses to nighttime temperature in Arabidopsis studied by genomic prediction

Ana Carolina dos Santos Sá, Alina Bazarova, Andreas Fischbach, Stefan Kesselheim, Benjamin Stich, and Shizue Matsubara

Supporting Information S1

Table S1. List of 308 *Arabidopsis thaliana* accessions.

Ecotype ID refers to the accession identification in the genotype data. Country refers to the origin of the accession.

Accession	Ecotype ID	Country
11ME1.32	8610	United States of America
11PNA4.101	8796	United States of America
328PNA054	8692	United States of America
627ME-4Y1	—	United States of America
Aa-0	7000	Germany
Ag-0	6897	France
Alc-0	6988	Spain
ALL1-2	1	France
Amel-1	6990	Netherlands
An-1	6898	Belgium
An-2	6996	Belgium
Ann-1	6994	France
App1-16	5832	Sweden
Ba-1	7014	United Kingdom
Ba1-2	8256	Sweden
Baa-1	7002	Netherlands
Bay-0	6899	Germany
Be-1	7011	Germany
Belmonte-4-94	957	Italy
Benk-1	7008	Netherlands
Bg-2	6709	United States of America
Bla-1	8264	Spain
Blh-1	8265	Czech Republic
Blh-2	7035	Czech Republic
Boot-1	7026	United Kingdom
Bor-4	6903	Czech Republic
Br-0	6904	Czech Republic
Bro1-6	8231	Sweden
Bs-2	7004	Switzerland
Bsch-0	7031	Germany
BUI	—	France
Bu-0	8271	Germany
Bur-0	6905	Ireland
C24	6906	Portugal
Ca-0	7062	Germany
CAM-16	23	France
CAM-61	66	France
Can-0	8274	Spain
Cen-0	8275	France
Cha-0	7069	Switzerland

Chat-1	7071	France
CIBC-17	6907	United Kingdom
CIBC2	6727	United Kingdom
CIBC4	6729	United Kingdom
CIBC5	6730	United Kingdom
Cit-0	7075	France
CLE-6	—	France
Co-2	7078	Portugal
Co-4	7080	Portugal
Col-0	6909	United States of America
CSHL-5	6744	United States of America
Ct-1	6910	Italy
CUR-3	81	France
Cvi-0	6911	Cape Verde
Da-0	7094	Germany
Db-0	7100	Germany
Di-1	7098	France
Do-0	7102	Germany
DraIV1-14	5896	Czech Republic
DraIV1-5	5887	Czech Republic
DraIV1-7	—	Czech Republic
DraIV6-16	5987	Czech Republic
DraIV6-35	6005	Czech Republic
Duk	6008	Czech Republic
Ede-1	7110	Netherlands
Eden-2	6913	Sweden
Edi-0	6914	United Kingdom
Ep-0	7123	Germany
Es-0	7126	Finland
Est-0	7128	Russia
Est-1	6916	Russia
Fab-4	6918	Sweden
Fei-0	8215	Portugal
Fi-1	7139	Germany
Fja1-2	6019	Sweden
Fja1-5	6020	Sweden
Fr-4	7135	Germany
Ga-0	6919	Germany
Ga-2	7141	Germany
Gd-1	8296	Germany
Ge-0	8297	Switzerland
Ge-1	7145	Switzerland
Gel-1	7143	Netherlands
Gie-0	7147	Germany

Gö-0	7151	Germany
Got-7	6921	Germany
Gr-1	8300	Austria
Gul1-2	—	Sweden
Gy-0	8214	France
Ha-0	7163	Germany
Hau-0	7164	Denmark
Hey-1	7166	Netherlands
Hi-0	8304	Netherlands
Hn-0	7165	Germany
Hod	8235	Czech Republic
Hov4-1	8306	Sweden
Hovdala-2	6039	Sweden
HR-5	6924	United Kingdom
Hs-0	8310	Germany
HSm	8236	Czech Republic
In-0	8311	Austria
JEA	91	France
Jl-3	7424	Czech Republic
Ka-0	8314	Austria
Kas-2	—	India
KBS-Mac-8	1716	United States of America
Kelsterbach-2	7188	Germany
Kelsterbach-4	8420	Germany
Kin-0	6926	United States of America
Kl-5	7199	Germany
Kn-0	7186	Lithuania
Kno-18	6928	United States of America
Koln	8239	Germany
Kr-0	7201	Germany
Kro-0	7206	Germany
Krot-2	7205	Germany
Kulturen-1	8240	Sweden
LAC-3	94	France
LAC-5	96	France
LDV-14	104	France
LDV-25	116	France
LDV-58	149	France
Li-3	7224	Germany
Li-5:2	7227	Germany
Li-7	7231	Germany
Liarum	8241	Sweden
Lillo-1	8242	Sweden
LI-OF-095	641	United States of America

Lip-0	8325	Poland
Lis-1	8326	Sweden
Lis-2	8222	Sweden
Lisse	8430	Netherlands
Lm-2	8329	France
Lom1-1	6042	Sweden
Lov-5	6046	Sweden
Lp2-2	7520	Czech Republic
Lp2-6	7521	Czech Republic
Lund	8335	Sweden
Lz-0	6936	France
Map-42	2057	United States of America
Mc-0	7252	United Kingdom
Mh-0	7255	Poland
MIB-15	166	France
MIB-22	173	France
MIB-28	178	France
MIB-84	223	France
MNF-Che-2	1925	United States of America
MNF-Jac-32	1967	United States of America
MNF-Pot-48	1859	United States of America
MNF-Pot-68	1867	United States of America
Mnz-0	7244	Germany
MOG-37	242	France
Mr-0	7522	Italy
Mrk-0	6937	Germany
Mt-0	6939	Libya
Mz-0	6940	Germany
N13	7438	Russia
N4	7446	Russia
Na-1	8343	France
Nc-1	7430	France
NC-6	—	United States of America
Nd-1	6942	Switzerland
NFA-10	6943	United Kingdom
NFA-8	6944	United Kingdom
No-0	7275	Germany
Nok-1	7270	Netherlands
Nw-0	7258	Germany
Nw-2	7260	Germany
Nz1	7263	New Zealand
Old-1	7280	Germany
Omo2-1	7518	Sweden
Or-0	7282	Germany

Or-1	6074	Sweden
Ors-1	7283	Romania
Ors-2	7284	Romania
Ost-0	8351	Sweden
Oy-0	6946	Norway
Pa-2	7291	Italy
PAR-3	258	France
PAR-4	259	France
PAR-5	260	France
Paw-3	2150	United States of America
Pent-1	2187	United States of America
Per-1	8354	Russia
Petergof	7296	Russia
PHW-10	7479	United Kingdom
PHW-13	7482	United Kingdom
PHW-14	7483	United Kingdom
PHW-20	7490	United Kingdom
PHW-26	7496	United Kingdom
PHW-28	7498	United Kingdom
PHW-33	7504	Netherlands
PHW-34	—	France
PHW-35	7506	France
PHW-37	7508	France
Pn-0	7307	France
Pna-17	7523	United States of America
Pro-0	8213	Spain
Pu2-23	6951	Czech Republic
PUZ24	—	—
Ra-0	6958	France
Rak-2	8365	Czech Republic
Ren-1	6959	France
Rev-2	6076	Sweden
Rhen-1	7316	Netherlands
Rmx-A180	7525	United States of America
ROM-1	267	France
RRS	—	—
Rsch-4	8374	Russia
S96	7472	Netherlands
Sanna-2	8376	Sweden
Sap-0	8378	Czech Republic
Sapporo-0	7330	Japan
Sav-0	7340	Czech Republic
Sav-1	—	—
Se-0	6961	Spain

Sg-1	7344	Germany
Shahdara	6962	Tajikistan
Si-0	7337	Germany
SLSP-30	2274	United States of America
Sp-0	7343	Germany
Sq-8	6967	United Kingdom
St-0	8387	Sweden
Ste-0	7346	Germany
Ste-3	2290	United States of America
T1040	6094	Sweden
T1060	6096	Sweden
T1080	6098	Sweden
T1110	6100	Sweden
T1130	6102	Sweden
T540	6112	Sweden
T690	6124	Sweden
Tad01	6169	Sweden
TDr-1	6188	Sweden
TDr-18	6203	Sweden
TDr-3	6190	Sweden
TDr-8	6194	Sweden
Tha-1	7353	Netherlands
Tiv-1	7355	Italy
Tomegap-2	6242	Sweden
Tottarp-2	6243	Sweden
TOU-A1-115	281	France
TOU-A1-116	282	France
TOU-A1-12	—	France
TOU-A1-43	321	France
TOU-A1-62	328	France
TOU-A1-67	—	France
TOU-A1-96	357	France
TOU-C-3	362	France
TOU-E-11	366	France
TOU-H-12	373	France
TOU-H-13	374	France
TOU-I-17	378	France
TOU-I-2	379	France
TOU-I-6	380	France
TOU-J-3	383	France
TOU-K-3	386	France
Ts-1	6970	Spain
Tscha-1	7372	Austria
Tsu-0	7373	Japan

Ty-0	7351	United Kingdom
Udul1-34	6318	Czech Republic
Uk-1	7378	Germany
UKCW06202	—	United Kingdom
UKID22	5729	United Kingdom
UKID37	5742	United Kingdom
UKID48	5753	United Kingdom
UKNW06-059	5380	United Kingdom
UKNW06-060	5381	United Kingdom
UKNW06-386	5565	United Kingdom
UKNW06-436	5606	United Kingdom
UKNW06-460	5628	United Kingdom
UKSE06-062	4997	United Kingdom
UKSE06-192	5056	United Kingdom
UKSE06-272	5116	United Kingdom
UKSE06-349	5158	United Kingdom
UKSE06-351	5160	United Kingdom
UKSE06-429	5207	United Kingdom
UKSE06-466	5232	United Kingdom
UKSE06-482	5245	United Kingdom
UKSE06-520	5264	United Kingdom
UKSE06-628	5341	United Kingdom
Ull2-3	6973	Sweden
Ull2-5	6974	Sweden
Ull3-4	6413	Sweden
Uod-7	6976	Austria
Utrecht	7382	Netherlands
Van-0	6977	Canada
Var2-1	7516	Sweden
VOU-1	390	France
VOU-2	392	France
Wa-1	7394	Poland
Wag-3	7390	Netherlands
Wag-4	7391	Netherlands
Wag-5	7392	Netherlands
Wc-2	7405	Germany
Wei-0	6979	Switzerland
Wil-1	—	Lithuania
Wl-0	7411	Germany
Ws	7397	Russia
Ws-0	6980	Russia
Wt-3	7408	Germany
Wt-5	6982	Germany
Yo-0	6983	United States of America

Zdr-6	6985	Czech Republic
Zdrl2-24	6448	Czech Republic
Zdrl2-25	6449	Czech Republic
Zu-1	7418	Switzerland

Table S2. List of nine accessions in the validation experiment.

Ecotype ID refers to the accession identification in the genotype data. Alongside these accessions, Col-0 was included as reference in the validation experiment.

Accession	Ecotype ID	Country
CUR-8	86	France
PAR-8	262	France
Paw-4	2151	United States of America
Kyl-1	5751	United Kingdom
DraIV3-8	5922	Czech Republic
Ei-2	6915	Germany
LL-0	6933	Spain
Bs-1	8270	Switzerland
Sim-1	9442	Sweden

Table S3. Hyperparameters evaluated in the grid search to maximize the predictive performance of XGBoost.

Hyperparameter	Values
Maximal depth of a tree	3, 5, 6, 10
Learning rate	0.0005, 0.005, 0.05, 0.01
Subsampling of training set	0.8, 0.9
Number of estimators	100, 500

Table S4. Variability in the effective (F_q'/F_m') and the maximal efficiency (F_v/F_m) of photosystem II, projected rosette area, and relative growth rate (RGR) among replicates of Col-0. N, number of evaluated individuals; SD, standard deviation. Means are observed (non-adjusted) means. RGR was calculated from the projected rosette area measured on two consecutive days. For example, RGR on 16 days after stratification (DAS) was calculated from the projected rosette area measured on 16 and 17 DAS.

Trait	T_{night}	DAS	N	Mean	SD
F_q'/F_m'	15°C	16	6	0.45	0.021
		17	6	0.47	0.016
		18	6	0.48	0.012
	20°C	16	10	0.47	0.010
		17	10	0.48	0.009
		18	10	0.48	0.010
F_v/F_m	15°C	16	6	0.77	0.010
		17	6	0.78	0.011
		18	6	0.79	0.003
	20°C	16	10	0.77	0.007
		17	10	0.77	0.005
		18	10	0.78	0.005
Projected rosette area (cm^2)	15°C	16	6	0.89	0.41
		17	6	1.27	0.61
		18	6	1.86	0.86
	20°C	16	10	1.87	0.76
		17	10	2.72	0.96
		18	10	4.04	1.45
RGR (% d^{-1})	15°C	16	6	33.8	8.04
		17	6	38.4	6.91
	20°C	16	10	39.5	5.84
		17	10	39.3	3.00

Table S5. Descriptive statistics of the adjusted entry means of the effective (F_q'/F_m') and the maximal efficiency (F_v/F_m) of photosystem II, projected rosette area, and relative growth rate (RGR).

Adjusted entry means calculated for individual accessions were averaged across all accessions (Mean ± 95% confidence interval, CI). Minimum and maximum are the minimal and the maximal values of adjusted entry means found in the accessions. RGR was calculated from the projected rosette area measured on two consecutive days. For example, RGR on 16 days after stratification (DAS) was calculated from the projected rosette area measured on 16 and 17 DAS.

Trait	T_{night}	DAS	Mean ± CI	Minimum	Maximum
F_q'/F_m'	15°C	16	0.43±0.003	0.33	0.48
		17	0.44±0.003	0.31	0.49
		18	0.45±0.002	0.34	0.50
	20°C	16	0.45±0.002	0.33	0.49
		17	0.46±0.002	0.36	0.50
		18	0.47±0.002	0.38	0.51
F_v/F_m	15°C	16	0.77±0.001	0.69	0.79
		17	0.77±0.001	0.65	0.80
		18	0.78±0.001	0.71	0.80
	20°C	16	0.76±0.001	0.72	0.78
		17	0.77±0.001	0.69	0.79
		18	0.77±0.001	0.71	0.79
Projected rosette area (cm^2)	15°C	16	0.59±0.02	0.25	1.07
		17	0.85±0.02	0.15	1.60
		18	1.25±0.03	0.23	2.39
	20°C	16	1.20±0.03	0.68	2.35
		17	1.76±0.04	0.83	3.27
		18	2.54±0.06	1.20	4.87
RGR (% d^{-1})	15°C	16	38.3±0.58	23.1	50.6
		17	38.5±0.47	24.5	50.9
	20°C	16	38.9±0.38	30.4	48.2
		17	37.4±0.34	28.0	44.7

Table S6. Variability in the adjusted entry means and model residuals among plant replicates for the effective (F_q'/F_m') and the maximal efficiency (F_v/F_m) of photosystem II, projected rosette area, and relative growth rate (RGR).

Standard deviation (SD) of adjusted entry means (AEM) among plant replicates of each accession was averaged across all accessions and is shown together with the maximal and the minimal SD values found among the accessions. The models' residuals were obtained for individual accessions and averaged across all accessions to represent the remaining variation in the phenotype of plant replicates after accounting for random factors in the linear mixed models. The SDs of the residuals thus calculated are also shown together with the maximal and the minimal SD values. DAS, days after stratification; N, number of evaluated accessions.

Trait	T_{night}	DAS	N	Mean SD of AEM	Maximal SD of AEM	Minimal SD of AEM	Mean model residuals	Mean SD of residuals	Maximal SD of residuals	Minimal SD of residuals
F_q'/F_m'	15°C	16	307	0.0013	0.0016	0.0012	5.45e-18	0.0007	0.0018	5.77e-06
		17	308	0.0013	0.0017	0.0012	-3.11e-18	0.0007	0.0025	9.77e-06
		18	308	0.0013	0.0016	0.0012	5.61e-19	0.0007	0.0018	2.83e-05
	20°C	16	307	0.0011	0.0015	0.0010	-1.18e-18	0.0006	0.0023	5.95e-06
		17	308	0.0011	0.0016	0.0010	2.37e-18	0.0006	0.0021	2.28e-05
		18	308	0.0011	0.0015	0.0010	-5.93e-18	0.0006	0.0021	6.79e-06
F_v/F_m	15°C	16	307	0.0003	0.0004	0.0002	-9.29e-19	0.0001	0.0004	2.10e-06
		17	308	0.0003	0.0004	0.0003	5.19e-19	0.0001	0.0006	1.09e-06
		18	308	0.0003	0.0003	0.0003	5.70e-19	0.0001	0.0005	3.40e-06
	20°C	16	307	0.0002	0.0002	0.0001	5.24e-19	0.0001	0.0004	6.52e-06
		17	308	0.0002	0.0003	0.0001	4.11e-19	0.0001	0.0003	1.42e-06
		18	308	0.0001	0.0002	0.0001	-7.02e-20	0.0001	0.0003	2.10e-07
Projected rosette area (cm^2)	15°C	16	307	0.537	0.690	0.486	-7.09e-16	0.284	0.897	0.001
		17	308	0.528	0.674	0.479	-1.26e-15	0.285	1.034	0.003
		18	308	0.515	0.654	0.468	1.13e-15	0.280	0.995	0.001
	20°C	16	307	0.560	0.934	0.489	-2.86e-15	0.204	0.985	0.000
		17	308	0.537	0.896	0.469	-5.68e-15	0.197	1.013	0.006
		18	308	0.520	0.867	0.453	1.42e-15	0.186	0.960	0.001

RGR (% d^{-1})	15°C	16	307	7.121	8.461	6.959	-6.69e-14	4.571	13.181	0.028
		17	308	6.008	6.528	5.862	1.14e-14	4.747	14.069	0.021
	20°C	16	307	5.654	7.410	5.469	-5.72e-14	3.397	10.174	0.027
		17	308	5.064	6.436	4.857	-1.05e-14	3.388	10.565	0.093

Table S7. Single nucleotide polymorphisms (SNPs) associated with long-term response of F_q'/F_m' to night temperature.

The SNP-trait associations were considered significant when $-\log_{10}(P) \geq$ FDR (false discovery rate) threshold (0.05) and/or $-\log_{10}(P) > 4.0$ on at least two days. No. refers to the SNP number shown in Figure 2. Allele 1/2 shows the major/minor allele. The allele effects were estimated for the minor alleles and given in the unit of F_q'/F_m'. The r^2 values correspond to the proportions of the variance explained by individual SNPs or sets of SNPs (simultaneous fit). For pairs of SNPs that were in linkage disequilibrium (underlined), only one of them (in parentheses) was included in the simultaneous fit. DAS, days after stratification; MAF, minor allele frequency. *SNPs are located in intergenic regions.

No.	SNP	T_{night}	DAS	Allele 1/2	Col-0 allele	MAF (%)	$-\log_{10}$ (P)	Effect	r^2 (%)	Locus	Gene annotation
1	<u>Chr2_1138854</u>	15°C	16	C/T	C	12.33	4.63	-0.009	8.77	AT2G03730	ACT DOMAIN REPEAT 5
2	<u>(Chr2_1138985)</u>	15°C	16	C/T	C	11.99	5.14	-0.010	9.73	AT2G03730	ACT DOMAIN REPEAT 5
3	Chr3_6651631	15°C	16	A/C	A	21.23	5.80	-0.008	9.64	*	*
4	Chr3_16153315	15°C	16	T/C	T	45.89	4.10	0.006	6.81	AT3G44560	FATTY ACID REDUCTASE 8
			Simultaneous fit						18.30		
5	Chr1_846217	15°C	17	A/C	C	41.30	4.44	0.006	10.02	AT1G03410	2-oxoglutarate and Fe (II)-dependent oxygenase superfamily protein
6	Chr1_10444149	15°C	17	A/G	A	24.23	4.91	-0.007	7.02	AT1G29830	magnesium transporter CorA-like family protein
2	Chr2_1138985	15°C	17	C/T	C	11.95	4.07	-0.008	8.40	AT2G03730	ACT DOMAIN REPEAT 5
7	Chr3_1409314	15°C	17	T/C	T	2.39	6.54	-0.022	12.43	AT3G05050	protein kinase superfamily protein
3	Chr3_6651631	15°C	17	A/C	A	21.16	4.35	-0.007	7.97	*	*
8	<u>Chr3_9768774</u>	15°C	17	A/C	A	6.48	4.60	-0.011	8.24	*	*
9	<u>(Chr3_9774541)</u>	15°C	17	A/G	A	6.48	4.60	-0.011	8.18	*	*
4	Chr3_16153315	15°C	17	T/C	T	45.73	4.15	0.005	6.61	AT3G44560	FATTY ACID REDUCTASE 8
10	Chr4_7788139	15°C	17	C/T	C	12.97	4.88	-0.009	9.93	AT4G13400	2-oxoglutarate and Fe (II)-dependent oxygenase superfamily protein

11	Chr4_7788811	15°C	17	A/T	A	5.46	4.77	-0.012	9.21	AT4G13400	2-oxoglutarate and Fe (II)-dependent oxygenase superfamily protein
12	Chr5_3676362	15°C	17	T/A	T	13.99	4.64	-0.008	9.17	AT5G11490	adaptin family protein
13	Chr5_5649958	15°C	17	C/T	T	29.35	4.19	-0.006	4.97	AT5G17170	rubredoxin family protein
		Simultaneous fit							32.01		
5	Chr1_846217	15°C	18	A/C	C	41.30	4.40	0.006	9.96	AT1G03410	2-oxoglutarate and Fe (II)-dependent oxygenase superfamily protein
6	Chr1_10444149	15°C	18	A/G	A	24.23	4.69	-0.006	6.59	AT1G29830	magnesium transporter CorA-like family protein
1	Chr2_1138854	15°C	18	C/T	C	12.29	4.11	-0.008	7.87	AT2G03730	ACT DOMAIN REPEAT 5
2	(Chr2_1138985)	15°C	18	C/T	C	11.95	4.69	-0.008	9.16	AT2G03730	ACT DOMAIN REPEAT 5
7	Chr3_1409314	15°C	18	T/C	T	2.39	5.21	-0.018	9.93	AT3G05050	protein kinase superfamily protein
8	Chr3_9768774	15°C	18	A/C	A	6.48	4.30	-0.010	7.83	*	*
9	(Chr3_9774541)	15°C	18	A/G	A	6.48	4.30	-0.010	7.58	*	*
10	Chr4_7788139	15°C	18	C/T	C	12.97	5.02	-0.008	9.74	AT4G13400	2-oxoglutarate and Fe (II)-dependent oxygenase superfamily protein
11	Chr4_7788811	15°C	18	A/T	A	5.46	4.37	-0.011	7.90	AT4G13400	2-oxoglutarate and Fe (II)-dependent oxygenase superfamily protein
12	Chr5_3676362	15°C	18	T/A	T	13.99	5.09	-0.008	10.28	AT5G11490	adaptin family protein
13	Chr5_5649958	15°C	18	C/T	T	29.35	4.21	-0.006	5.01	AT5G17170	rubredoxin family protein
		Simultaneous fit							29.09		
14	Chr1_13939977	20°C	16	A/G	G	31.85	7.88	-0.004	6.63	*	*
2	Chr2_1138985	20°C	16	C/T	C	11.99	8.54	-0.007	11.37	AT2G03730	ACT DOMAIN REPEAT 5
15	Chr2_11170979	20°C	16	A/G	A	2.05	9.63	-0.015	18.17	AT2G26250	3-KETOACYL-COA SYNTHASE 10

16	Chr3_3946441	20°C	16	C/T	C	16.78	5.40	-0.004	9.00	AT3G12410	polynucleotidyl transferase
17	Chr4_7156570	20°C	16	A/T	T	11.30	7.98	-0.006	2.62	AT4G11910	STAY-GREEN-like protein
18	Chr4_7653545	20°C	16	G/T	G	31.16	7.54	0.004	3.82	*	*
19	Chr4_8562879	20°C	16	T/G	T	13.36	6.53	-0.005	9.25	AT4G14980	cysteine/histidine-rich C1 domain family protein
20	Chr5_14578542	20°C	16	C/A	C	41.44	6.91	-0.004	6.49	AT5G36935	transposable element
21	Chr5_15330192	20°C	16	G/T	T	41.44	7.05	0.004	4.22	AT5G38350	disease resistance protein (NBS-LRR class) family
		Simultaneous fit							39.87		
22	Chr1_2029747	20°C	17	A/T	T	47.78	9.00	-0.004	4.88	AT1G06630	F-box/RNI-like superfamily protein
23	Chr1_8764728	20°C	17	C/T	C	44.71	6.76	-0.003	3.01	*	*
15	Chr2_11170979	20°C	17	A/G	A	2.05	23.59	-0.024	19.09	AT2G26250	3-KETOACYL-COA SYNTHASE 10
16	Chr3_3946441	20°C	17	C/T	C	16.72	7.06	-0.004	10.05	AT3G12410	polynucleotidyl transferase
24	Chr4_2580731	20°C	17	G/A	A	30.38	6.07	-0.003	1.62	AT4G05040	ankyrin repeat family protein
25	Chr4_11273457	20°C	17	G/A	G	5.80	8.95	-0.008	8.68	AT4G21120	AMINO ACID TRANSPORTER 1
26	Chr4_11827105	20°C	17	T/C	T	23.21	7.19	0.004	3.62	*	*
20	Chr5_14578542	20°C	17	C/A	C	41.30	8.57	-0.004	6.50	AT5G36935	transposable element
27	Chr5_18289242	20°C	17	A/G	G	24.57	9.49	-0.004	7.02	AT5G45200	disease resistance protein (TIR-NBS-LRR class) family
		Simultaneous fit							39.91		
28	Chr1_18957084	20°C	18	G/A	G	6.14	7.25	-0.007	11.19	*	*
29	Chr2_8145104	20°C	18	A/G	G	21.16	6.01	0.003	9.16	AT2G18800	XYLOGLUCAN ENDOTRANSGLUCOSYLASE/ HYDROLASE 21
15	Chr2_11170979	20°C	18	A/G	A	2.05	9.92	-0.015	17.75	AT2G26250	3-KETOACYL-COA SYNTHASE 10

30	Chr3_3218698	20°C	18	G/A	G	5.46	6.21	-0.006	2.02	AT3G10370	FAD-dependent oxidoreductase family protein
31	Chr3_9123175	20°C	18	G/C	G	20.14	8.57	-0.004	6.71	AT3G25030	RING/U-box superfamily protein
32	Chr4_7274303	20°C	18	G/T	T	38.91	11.24	-0.004	8.14	AT4G12170	Thioredoxin superfamily protein
33	Chr4_11273410	20°C	18	G/C	G	6.14	10.41	-0.008	9.29	AT4G21120	AMINO ACID TRANSPORTER 1
34	Chr5_9769302	20°C	18	T/C	T	40.27	7.50	-0.003	2.90	AT5G27610	ALWAYS EARLY 1
35	Chr5_16900781	20°C	18	G/T	G	46.08	6.49	-0.003	1.86	*	*
27	Chr5_18289242	20°C	18	A/G	G	24.57	12.89	-0.005	8.63	AT5G45200	disease resistance protein (TIR-NBS-LRR class) family
		Simultaneous fit							45.58		

Table S8. Number of single nucleotide polymorphisms (SNPs) used as predictors of F_q'/F_m'.

**T_{night}**	**DAS**	**$-\log_{10}$ (*P*) ≥ FDR and/or $-\log_{10}$ (*P*) > 4.0 on at least two days**	**$-\log_{10}$ (*P*) ≥ 3.0 on the respective day**
15°C	16	4	192
	17	12	231
	18	11	232
20°C	16	9	192
	17	9	160
	18	10	260

DAS, days after stratification; FDR, false discovery rate; T_{night}, nighttime temperature.

Table S9. Performance of gBLUP and Bayesian approaches in predicting F_q'/F_m' for the 293 Arabidopsis accessions in the GWAS panel.

F_q'/F_m' data of the 293 accessions were used to train prediction models based on 211,771 SNPs. Only on 16 days after stratification (DAS), one accession (T1080) was excluded. Predictive performance was assessed using root mean squared error (RMSE) and Pearson correlation coefficient (COR) averaged across three testing sets (± SE).

T_{night}	Method	DAS	RMSE	COR
15°C	gBLUP	16	0.022±0.000	0.20±0.13
	BayesA	16	0.022±0.000	0.19±0.13
	BayesB	16	0.022±0.000	0.19±0.12
	gBLUP	17	0.022±0.002	0.23±0.06
	BayesA	17	0.022±0.002	0.24±0.06
	BayesB	17	0.022±0.002	0.23±0.06
	gBLUP	18	0.021±0.002	0.23±0.02
	BayesA	18	0.021±0.002	0.23±0.01
	BayesB	18	0.021±0.002	0.23±0.01
20°C	gBLUP	16	0.022±0.001	0.15±0.09
	BayesA	16	0.022±0.001	0.17±0.08
	BayesB	16	0.022±0.001	0.15±0.07
	gBLUP	17	0.018±0.001	0.25±0.01
	BayesA	17	0.018±0.001	0.26±0.01
	BayesB	17	0.018±0.001	0.27±0.01
	gBLUP	18	0.016±0.000	0.21±0.03
	BayesA	18	0.016±0.001	0.23±0.02
	BayesB	18	0.016±0.001	0.23±0.02

gBLUP, genomic best linear unbiased prediction; T_{night}, night temperature.

Table S10. 47 SNPs identified by GWAS with -log10 (P) ≥ 3.0 on three consecutive days in 15°C T_{night}.

Shown are the associations of 47 SNPs with F_q'/F_m' on 16 and 17 days after stratification (DAS). The corresponding data for 18 DAS can be found in Table 1 together with locus and gene annotations. Allele 1/2 shows the major/minor allele. The allele effects were estimated for the minor alleles and given in the unit of F_q'/F_m'. The r^2 values give the proportion of the variance explained by individual SNPs or sets of SNPs (simultaneous fit). For pairs of SNPs that were in linkage disequilibrium (underlined), only one of them (in parentheses) was included in the simultaneous fit. MAF, minor allele frequency.

SNP	DAS	Allele 1/2	Col-0 allele	MAF (%)	-log10 (P)	Effect	r^2 (%)
Chr1_846217	16	A/C	C	41.44	4.00	0.006	9.65
Chr1_873447	16	A/T	A	34.25	3.41	-0.006	8.45
Chr1_884146	16	G/A	G	37.33	3.26	-0.005	7.24
Chr1_6478834	16	A/T	A	23.97	3.65	0.007	3.08
Chr1_10027869	16	T/C	T	9.25	3.56	0.009	7.27
Chr1_19515673	16	C/G	C	38.01	3.08	0.005	7.04
Chr1_23436110	16	G/C	G	6.85	3.58	-0.010	5.75
Chr2_1138854	16	C/T	C	12.33	4.63	-0.009	9.19
Chr2_1138985	16	C/T	C	11.99	5.14	-0.010	10.08
Chr2_1139010	16	A/C	A	14.73	4.46	-0.008	8.51
Chr2_2884161	16	C/T	T	31.16	3.01	-0.005	2.93
Chr2_8145104	16	A/G	G	21.23	3.62	0.007	7.17
Chr2_11170979	16	A/G	A	2.05	3.77	-0.019	8.72
Chr2_13684149	16	G/A	G	13.70	3.46	-0.007	6.67
Chr2_14059450	16	C/A	C	29.11	3.28	-0.006	6.99
Chr3_1275424	16	C/T	C	16.78	3.47	0.007	3.59
Chr3_3395109	16	C/G	C	9.59	3.18	0.009	6.94
Chr3_6651631	16	A/C	A	21.23	5.80	-0.008	10.12

Chr3_9636722	16	T/G	T	34.93	4.27	-0.006	8.09
Chr3_10582418	16	C/T	T	47.60	3.66	0.006	2.76
Chr3_10874763	16	T/A	A	39.73	3.20	-0.005	3.45
Chr3_10898637	16	G/A	G	28.77	4.11	-0.006	9.02
Chr3_16153315	16	T/C	T	45.89	4.10	0.006	6.70
Chr3_19054365	16	A/G	A	3.08	3.91	-0.016	8.34
Chr3_19257874	16	T/C	T	9.59	3.41	0.009	6.43
Chr3_21427556	16	G/A	G	12.67	4.89	-0.009	8.74
Chr3_21432797	16	C/T	C	21.23	4.87	-0.008	8.25
Chr3_21432851	16	C/T	C	21.92	4.62	-0.007	8.04
Chr3_22377074	16	A/G	A	36.64	3.70	-0.005	6.10
Chr4_5411416	16	C/G	C	19.18	3.22	-0.006	6.09
Chr4_5633114	16	C/A	C	3.08	4.30	-0.016	8.88
Chr4_7788139	16	C/T	C	12.67	3.43	-0.008	8.00
Chr4_14017514	16	C/T	C	15.07	3.48	-0.007	6.66
Chr4_14030315	16	T/G	T	16.10	3.10	-0.006	5.89
Chr4_15903731	16	C/G	C	2.05	3.75	-0.019	8.28
Chr4_18442418	16	A/G	A	12.67	3.90	-0.008	7.10
Chr5_700043	16	A/G	A	17.12	4.13	-0.008	5.20
Chr5_3284989	16	C/A	A	40.75	3.01	0.005	3.78
Chr5_5536626	16	A/C	A	19.18	3.11	-0.006	7.55
Chr5_5538353	16	G/A	G	18.15	3.22	-0.007	7.46
Chr5_5620806	16	C/T	T	46.58	3.99	-0.005	7.18
Chr5_5627597	16	T/G	T	29.79	3.24	-0.005	6.07
Chr5_5627893	16	A/G	G	35.62	3.38	-0.005	4.96
Chr5_9760768	16	C/A	C	6.16	3.74	-0.011	7.38
Chr5_18289242	16	A/G	G	24.32	3.21	-0.006	6.29
Chr5_21767983	16	T/C	T	3.08	3.93	-0.016	9.22

Chr5_23908100	16	C/A	A	46.58	3.04	0.005	6.64
			Simultaneous fit				49.09
Chr1_846217	17	A/C	C	41.30	4.44	0.006	10.25
Chr1_873447	17	A/T	A	34.47	3.79	-0.006	8.87
Chr1_884146	17	G/A	G	37.54	3.80	-0.005	7.32
Chr1_6478834	17	A/T	A	23.89	3.25	0.006	2.95
Chr1_10027869	17	T/C	T	9.22	3.09	0.008	6.43
Chr1_19515673	17	C/G	C	37.88	3.29	0.005	7.37
Chr1_23436110	17	G/C	G	6.83	3.01	-0.009	5.05
Chr2_1138854	17	C/T	C	12.29	3.67	-0.008	7.62
Chr2_1138985	17	C/T	C	11.95	4.07	-0.008	8.25
Chr2_1139010	17	A/C	A	14.68	3.35	-0.007	6.25
Chr2_2884161	17	C/T	T	31.40	3.56	-0.005	3.68
Chr2_8145104	17	A/G	G	21.16	4.05	0.007	8.09
Chr2_11170979	17	A/G	A	2.05	3.81	-0.018	8.14
Chr2_13684149	17	G/A	G	13.65	3.09	-0.006	6.02
Chr2_14059450	17	C/A	C	29.01	3.19	-0.005	6.72
Chr3_1275424	17	C/T	C	16.72	3.44	0.007	3.52
Chr3_3395109	17	C/G	C	9.56	3.05	0.008	7.12
Chr3_6651631	17	A/C	A	21.16	4.35	-0.007	8.13
Chr3_9636722	17	T/G	T	34.81	3.52	-0.005	7.25
Chr3_10582418	17	C/T	T	47.44	3.53	0.005	2.80
Chr3_10874763	17	T/A	A	39.93	3.86	-0.005	4.21
Chr3_10898637	17	G/A	G	28.67	3.29	-0.005	7.78
Chr3_16153315	17	T/C	T	45.73	4.15	0.005	6.59
Chr3_19054365	17	A/G	A	3.07	4.03	-0.015	9.16
Chr3_19257874	17	T/C	T	9.56	3.23	0.008	5.63
Chr3_21427556	17	G/A	G	12.63	3.82	-0.008	7.72

Chr3_21432797	17	C/T	C	21.16	3.41	-0.006	6.72
Chr3_21432851	17	C/T	C	21.84	3.28	-0.006	6.38
Chr3_22377074	17	A/G	A	36.86	3.43	-0.005	5.53
Chr4_5411416	17	C/G	C	19.11	3.25	-0.006	6.72
Chr4_5633114	17	C/A	C	3.07	3.63	-0.014	7.79
Chr4_7788139	17	C/T	C	12.97	4.88	-0.009	10.06
Chr4_14017514	17	C/T	C	15.36	4.03	-0.007	7.78
Chr4_14030315	17	T/G	T	16.38	3.57	-0.007	6.79
Chr4_15903731	17	C/G	C	2.05	3.96	-0.018	9.24
Chr4_18442418	17	A/G	A	12.63	3.26	-0.007	6.02
Chr5_700043	17	A/G	A	17.06	3.45	-0.006	4.43
Chr5_3284989	17	C/A	A	40.61	3.43	0.005	4.47
Chr5_5536626	17	A/C	A	19.11	3.25	-0.006	8.25
Chr5_5538353	17	G/A	G	18.09	3.10	-0.006	7.44
Chr5_5620806	17	C/T	T	46.76	4.60	-0.006	8.00
Chr5_5627597	17	T/G	T	30.03	3.53	-0.005	6.15
Chr5_5627893	17	A/G	G	35.84	3.51	-0.005	5.48
Chr5_9760768	17	C/A	C	6.14	4.58	-0.012	8.28
Chr5_18289242	17	A/G	G	24.57	3.58	-0.006	6.76
Chr5_21767983	17	T/C	T	3.07	3.57	-0.014	8.87
Chr5_23908100	17	C/A	A	46.42	3.59	0.005	7.12
			Simultaneous fit				47.08

Table S11. Performance of gBLUP in predicting F_q'/F_m' for the Arabidopsis GWAS panel in 15°C T_{night} based on four different sets of SNPs.

Predictors were 211,771 SNPs or the GWAS-derived SNPs with $-\log_{10}(P) \geq$ FDR threshold (0.05) and/or $-\log_{10}(P) > 4.0$ on at least two days (a), $-\log_{10}(P) \geq 3.0$ on the respective day (b), and 47 SNPs that satisfied $-\log_{10}(P) \geq 3.0$ on all three days (c). Predictive performance was assessed using root mean squared error (RMSE) and Pearson correlation coefficient (COR) averaged across three testing sets (± SE).

DAS	211,771 SNPs		FDR threshold[a]		$-\log_{10}(P) \geq 3.0$[b]		47 SNPs[c]	
	RMSE	COR	RMSE	COR	RMSE	COR	RMSE	COR
16	0.022±0.001	0.20±0.13	0.021±0.001	0.32±0.06	0.014±0.001	0.79±0.01	0.016±0.002	0.71±0.05
17	0.022±0.002	0.23±0.06	0.020±0.001	0.48±0.08	0.012±0.000	0.84±0.04	0.017±0.002	0.69±0.04
18	0.021±0.002	0.23±0.02	0.018±0.000	0.51±0.12	0.012±0.001	0.82±0.06	0.016±0.001	0.67±0.03

DAS, days after stratification; FDR, false discovery rate; T_{night}, night temperature.

Table S12. Performance of gBLUP in predicting F_q'/F_m' for the Arabidopsis GWAS panel in 20°C T_{night} on 18 days after stratification (DAS) based on SNPs identified in the same T_{night} but on non-matching days, or on the same day but in 15°C T_{night}.

For comparison, predictive performance based on the corresponding set of SNPs (*identified in 20°C T_{night} on 18 DAS) is also shown. Predictive performance was assessed using root mean squared error (RMSE) and Pearson correlation coefficient (COR) averaged across three test sets (± SE). The associations of all SNPs had $-\log_{10}(P) \geq 3.0$.

T_{night}	DAS	RMSE	COR
15°C	18	0.013±0.000	0.62±0.06
20°C	16	0.012±0.000	0.67±0.05
20°C	17	0.010±0.000	0.78±0.05
*20°C	18	0.008±0.001	0.86±0.03

Table S13. Performance of XGBoost in predicting F_q'/F_m' based on the genotype data adjusted and non-adjusted for population structure.

Prediction was based on 211,771 SNPs. Genotypes were adjusted for population structure using 250 principal components. Validation of the XGBoost model in the test set was performed using parameters selected in a grid search (see Table S3 for hyperparameters evaluated in the grid search). Predictive performance was assessed by root mean squared error (RMSE ±SE) averaged across three test sets.

T_{night}	DAS	RMSE	
		Non-adjusted	Adjusted
15°C	16	0.023±0.002	0.023±0.002
	17	0.023±0.003	0.022±0.003
	18	0.021±0.002	0.021±0.002
20°C	16	0.022±0.001	0.022±0.001
	17	0.017±0.002	0.018±0.001
	18	0.016±0.001	0.016±0.001

DAS, days after stratification; T_{night}, night temperature.

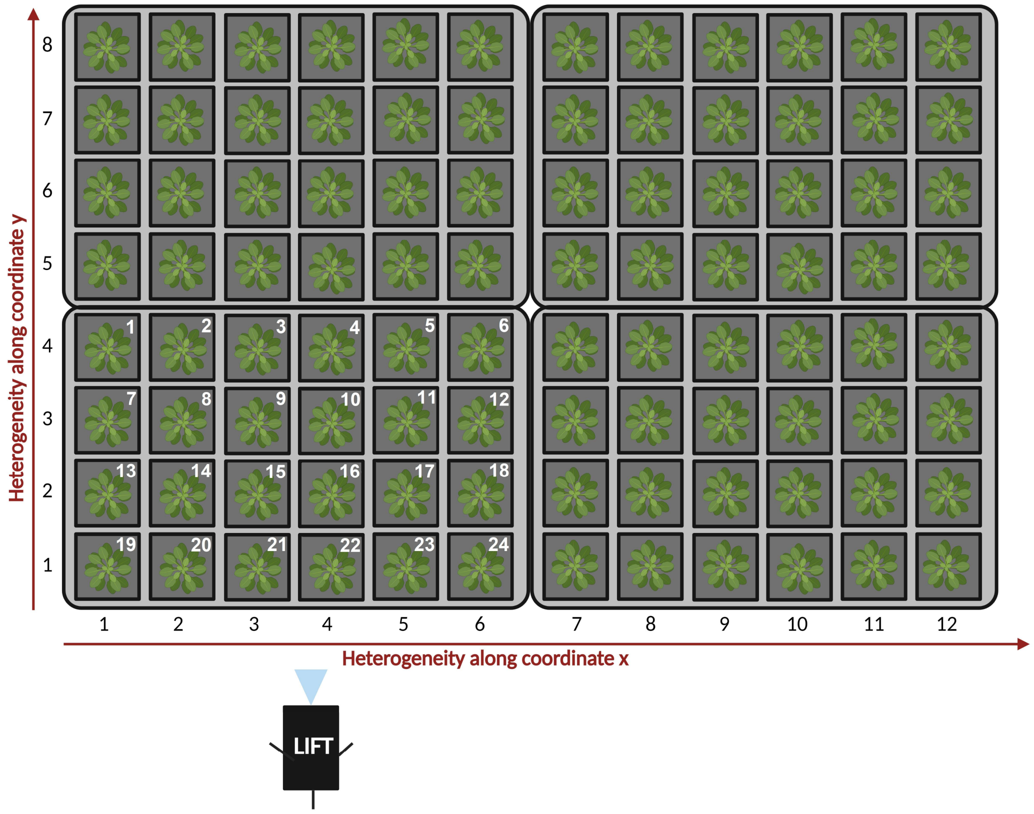

Figure S1. Scheme of plant positions in the inlets. Only four inlets are depicted as an example. To account for spatial heterogeneity of growth conditions (e.g. small differences in light intensity), two variables (coordinates x and y with numbering) were considered as random effects. For adjusting entry means of F_q'/F_m' and F_v/F_m measured by the LIFT instrument (indicated at the left bottom), the positions of plants inside the inlet (1-24, written in white) were considered as random effects.

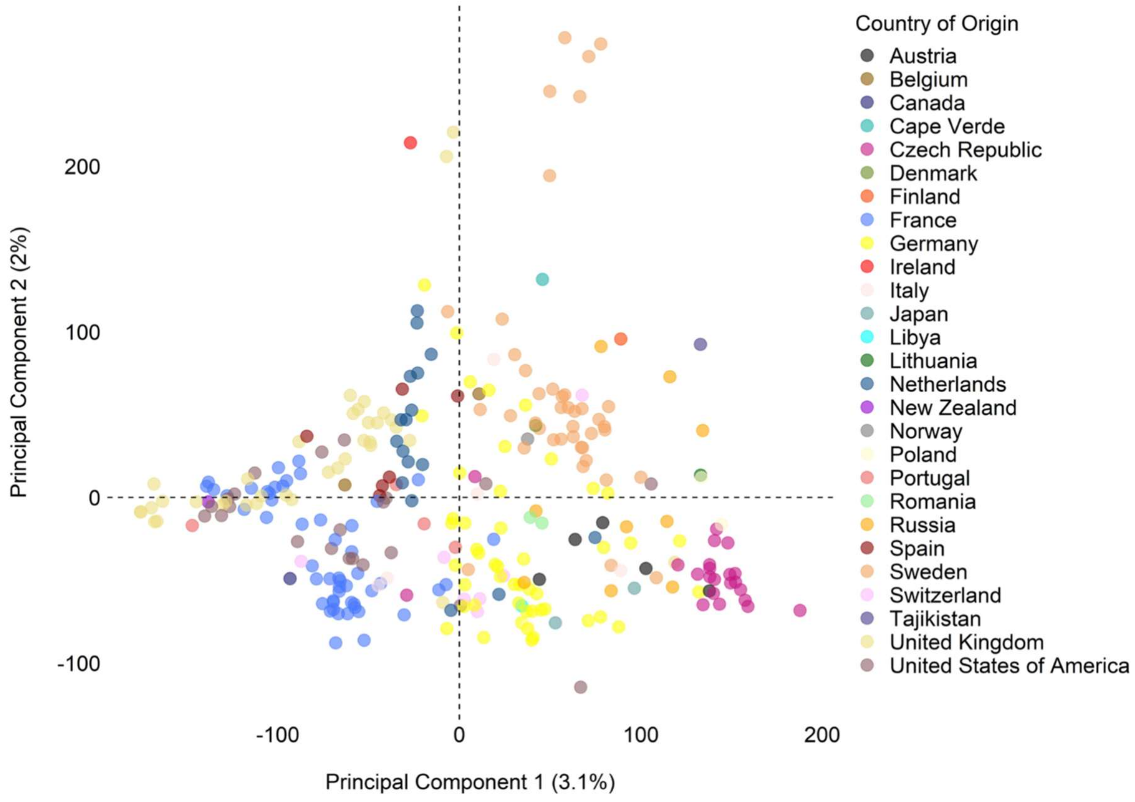

Figure S2. Population structure of the 293 Arabidopsis accessions. Principal component (PC) analysis was based on the 211,771 SNPs. Percentages of the total variance explained by PC1 and PC2 are 3.1% and 2%, respectively. Circles represent individual accessions (color-coded according to their country of origin).

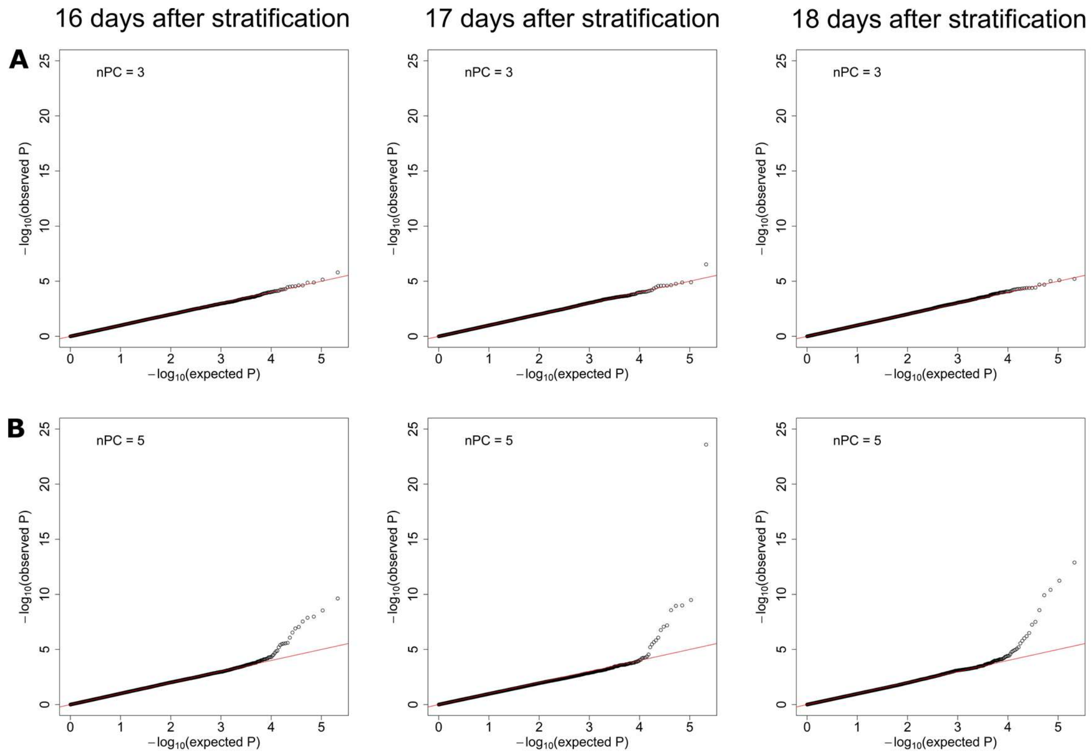

Figure S3. Q-Q plots of the observed and the expected *P* values distribution from the GWAS on F_q'/F_m' in the 293 accessions on 16, 17 and 18 days after stratification. A, 15°C T_{night}. B, 20°C T_{night}. nPC indicates the number of principal component included as covariates in FarmCPU.

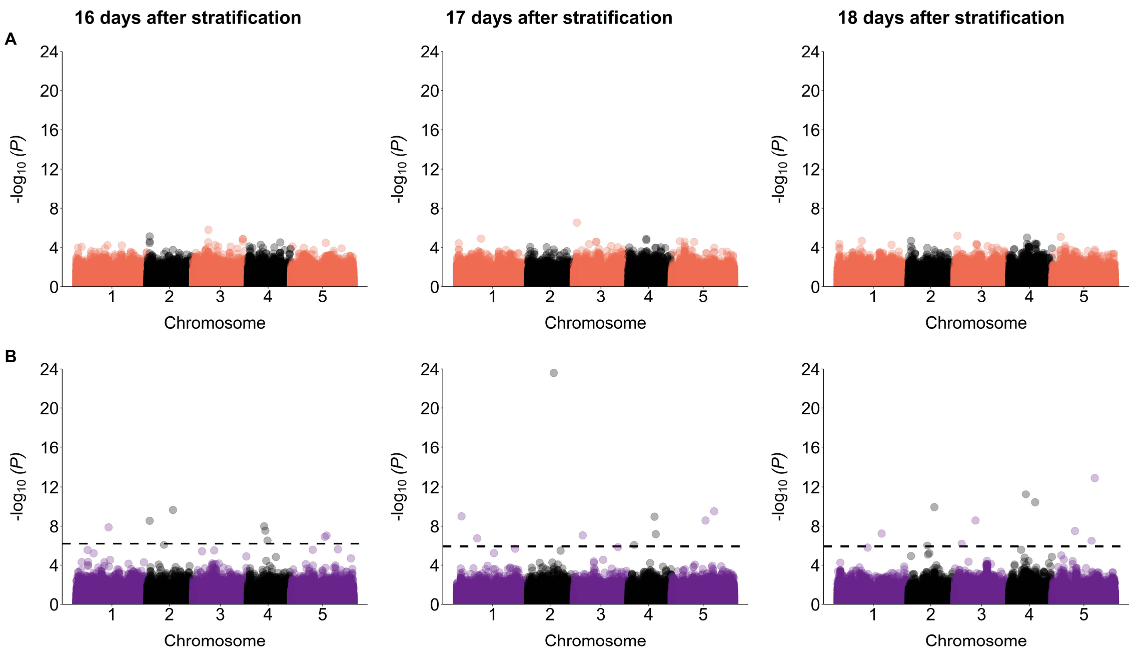

Figure S4. Manhattan plots of the GWAS on F_q'/F_m' in the 293 accessions. A, 15°C T_{night}. B, 20°C T_{night}. Dashed lines in (B) show the false discovery rate threshold (0.05).

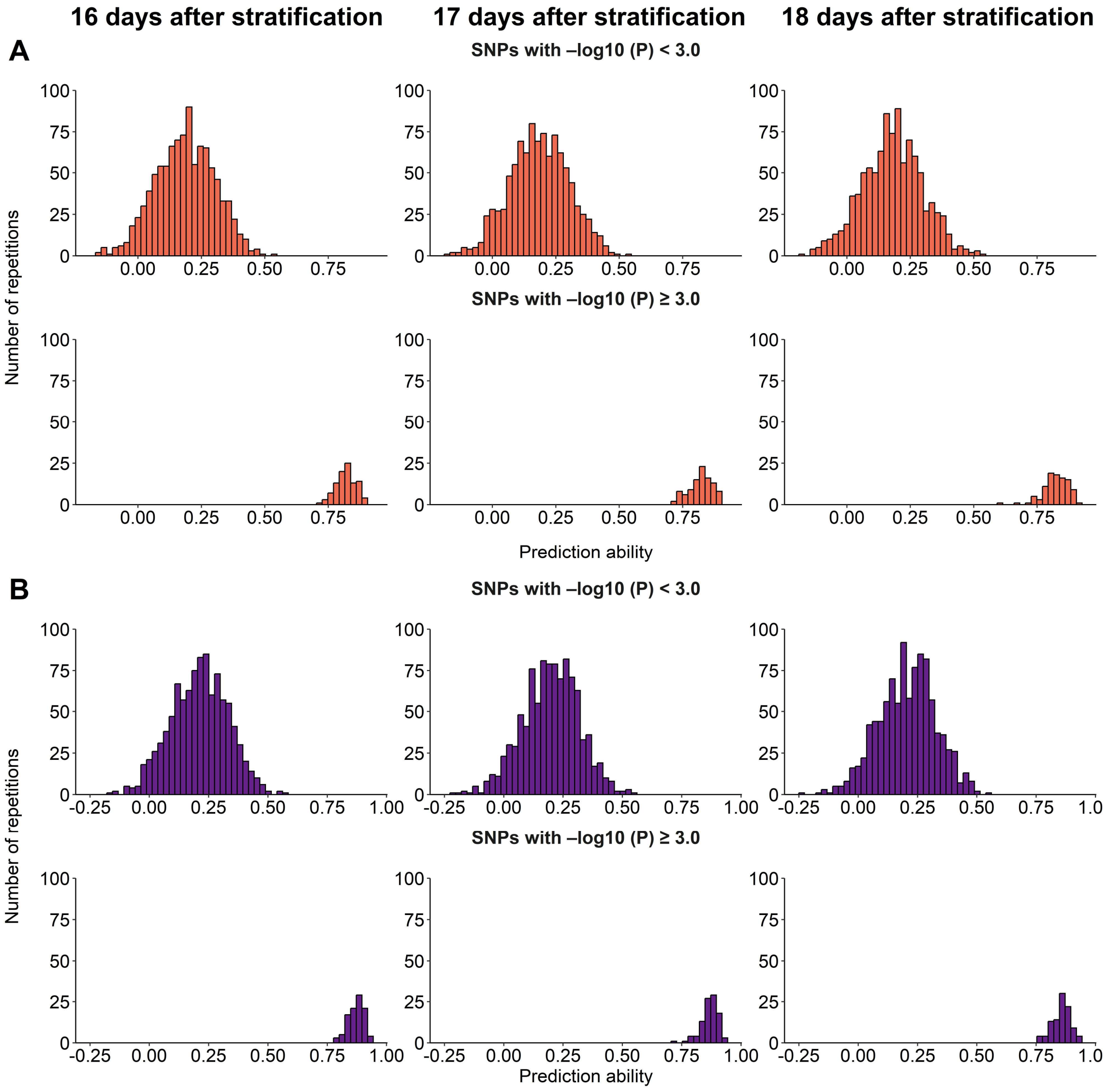

Figure S5. Distribution of prediction ability of gBLUP models for F_q'/F_m' based on the GWAS-derived SNPs with $-\log_{10}$ (P) < 3.0 or ≥ 3.0. A, 15°C T_{night}. B, 20°C T_{night}. For the plots of $-\log_{10}$ (P) < 3.0, prediction ability was calculated by randomly selecting the same number of SNPs as for $-\log_{10}$ (P) ≥ 3.0. Given the large number of SNPs with $-\log_{10}$ (P) < 3.0, prediction ability was calculated as the median across 992-1,000 replications.

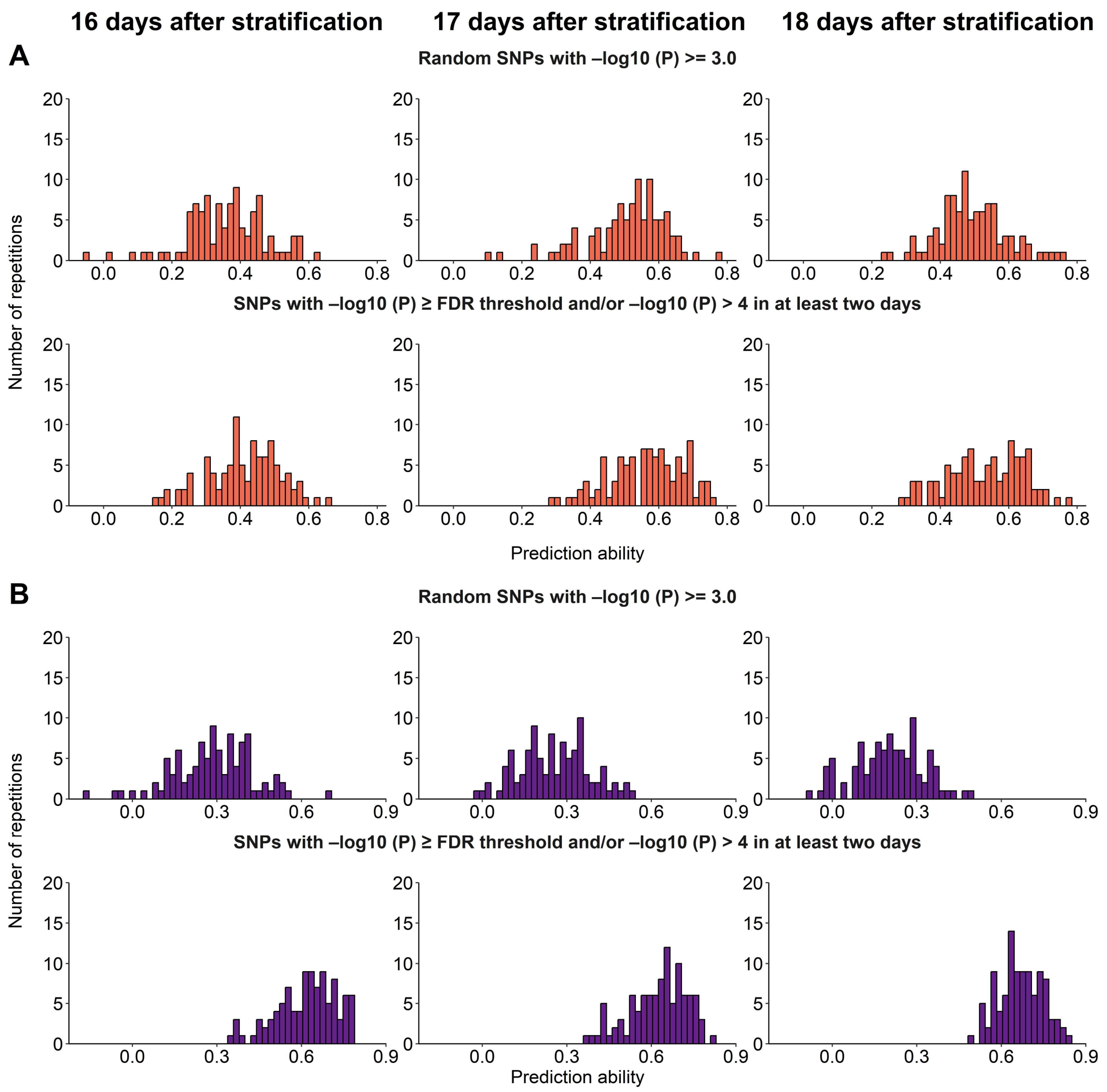

Figure S6. Distribution of prediction ability of gBLUP models for F_q'/F_m' based on random GWAS-derived SNPs with $-\log_{10}(P) \geq 3.0$ and SNPs with $-\log_{10}(P) \geq$ FDR threshold (0.05) and/or $-\log_{10}(P) > 4.0$ on at least two days. A, 15°C T_{night}. B, 20°C T_{night}. For the plots of random SNPs with $-\log_{10}(P) \geq 3.0$, prediction ability was calculated by randomly selecting the same number of SNPs as for $-\log_{10}(P) \geq$ FDR threshold and/or $-\log_{10}(P) > 4.0$ on at least two days.

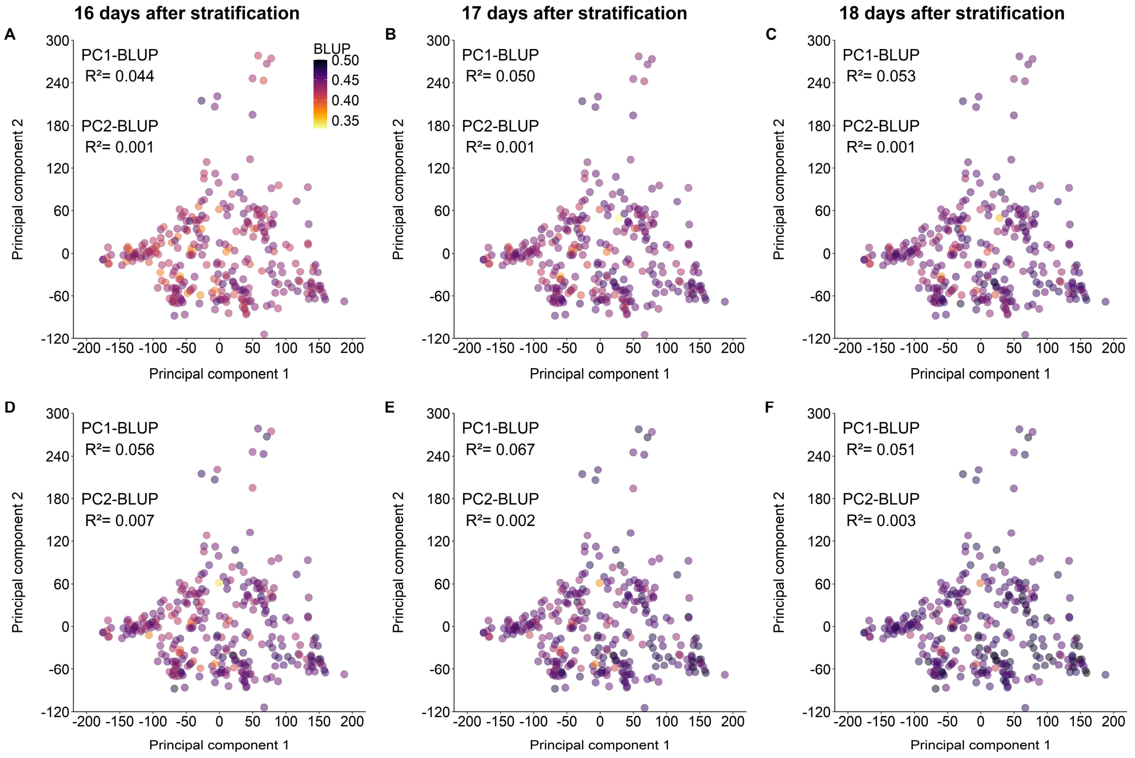

Figure S7. Population structure of the 293 accessions color-coded according to the F_q'/F_m' predicted by gBLUP. A-C, 15°C T_{night}. D-F, 20°C T_{night}. Principal component (PC) analysis was based on the 211,771 SNPs. Prediction of F_q'/F_m' was performed by gBLUP based on the GWAS-derived SNPs with $-\log_{10}(P) \geq 3.0$ on the respective day. The color scale in panel A shows coding scheme of the predicted F_q'/F_m'. R^2 indicates the correlation between PC1 or PC2 and the gBLUP prediction of F_q'/F_m'.

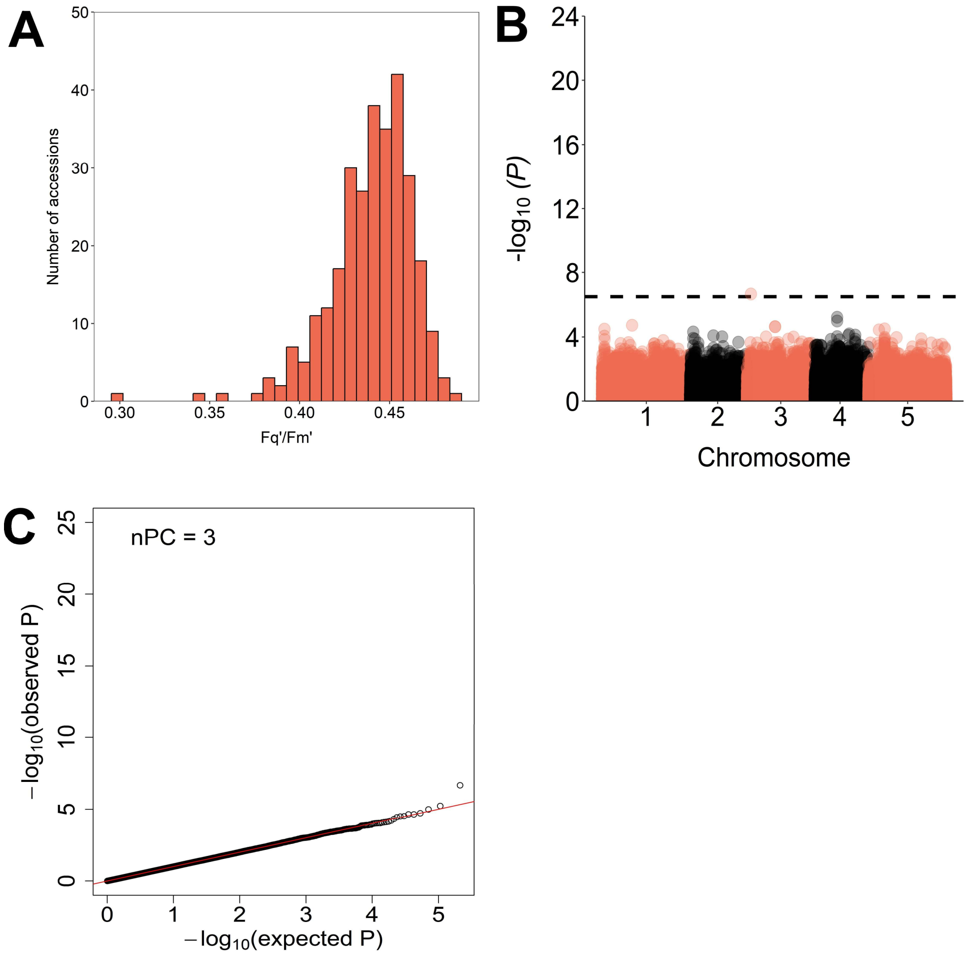

Figure S8. Analysis of F_q'/F_m' and its associated SNPs in 293 accessions in 15°C T_{night} across the three measurement days combined. A, Distribution of the adjusted entry means of F_q'/F_m' among the accessions; B, Manhattan plot; and C, Q-Q plot of the observed and the expected *P* values distribution. GWAS was performed on the adjusted entry means of F_q'/F_m' obtained by a linear mixed model using day as random effect. Dashed line in (B) shows the FDR (false discovery rate) threshold (0.05). nPC in (C) indicates the number of principal components included as covariates in FarmCPU.

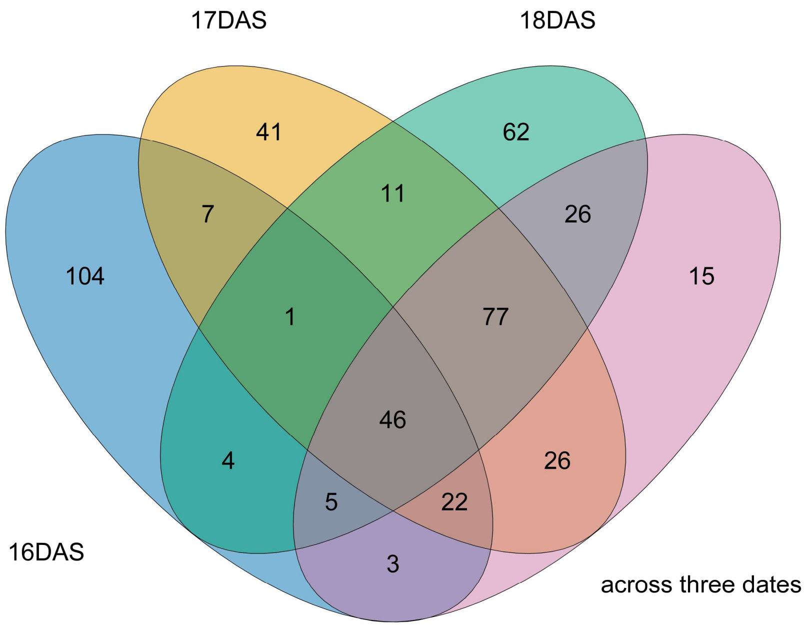

Figure S9. Shared and unique SNPs with -log$_{10}$ (P) ≥ 3.0 between the three measurement days analyzed separately and across the three days combined in the 15°C T_{night} condition.

.

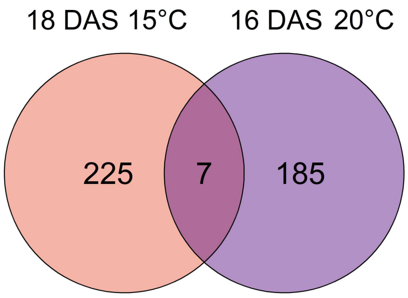

Figure S10. Shared and unique SNPs with $-\log_{10}(P) \geq 3.0$ between the plants growing in 15°C T_{night} on 18 days after stratification (DAS) and those growing in 20°C T_{night} on 16 DAS.

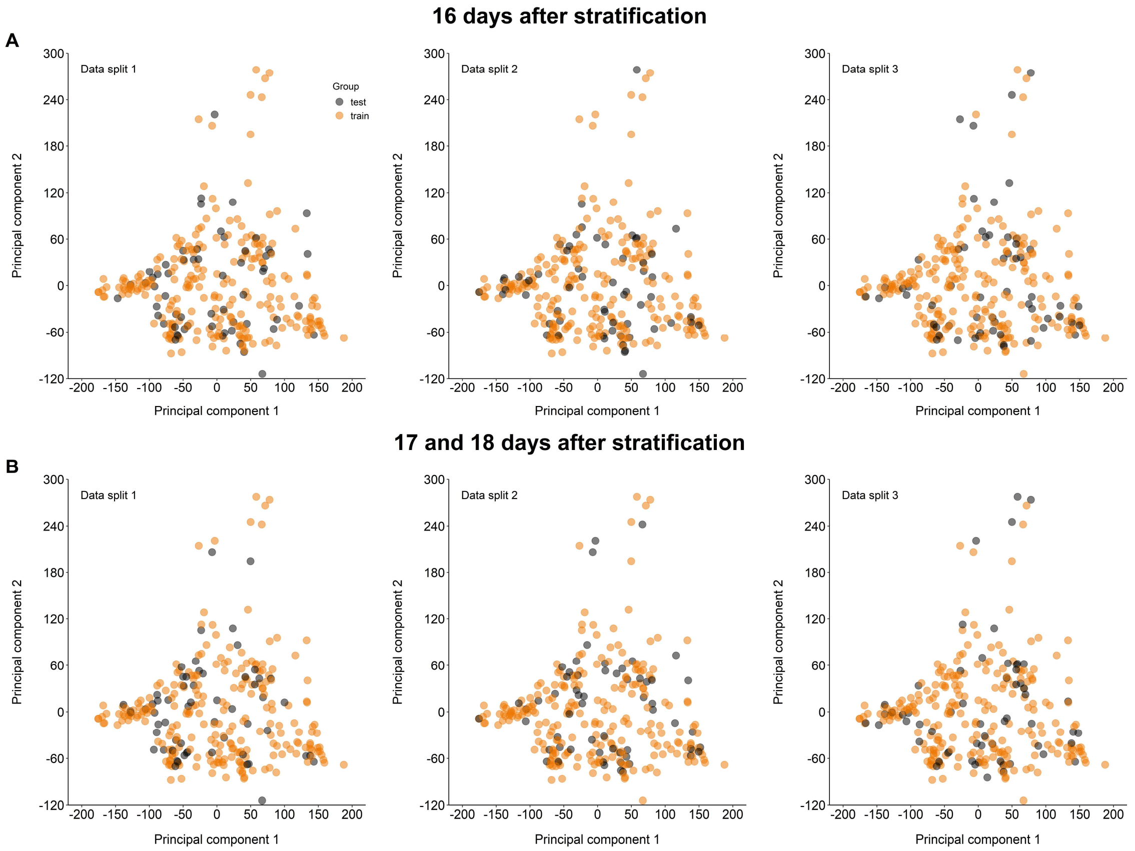

Figure S11. Population structure of randomly selected accessions for training and testing gBLUP models of F_q'/F_m'. The accessions were randomly split into training (orange) and test groups (black) at 4:1 ratio. The results of three test sets (three different splits) are shown for (A) 16 days after stratification (292 accessions without T1080) and (B) 17 and 18 days after stratification (all 293 accessions). Principal component analysis was based on the 211,771 SNPs.

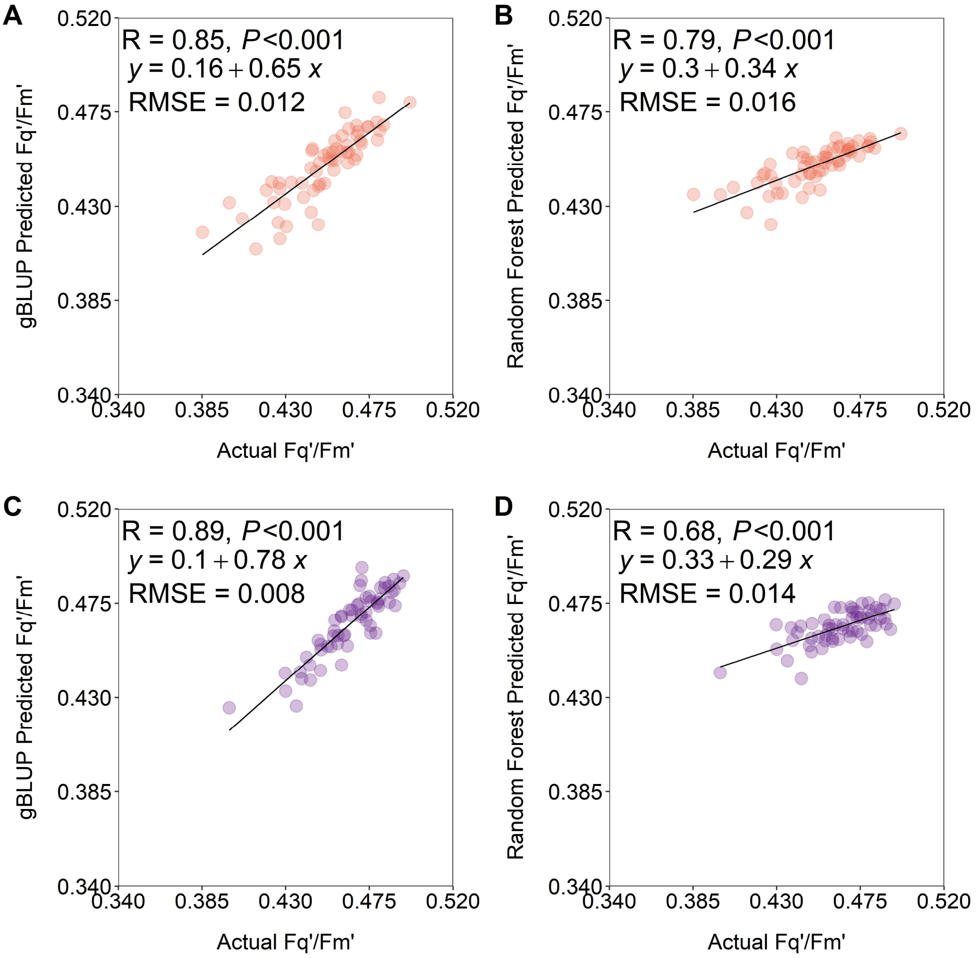

E

Tnight	Test set	Method	R	RMSE
15°C	2	gBLUP	0.91	0.010
		Random forest	0.83	0.017
20°C	2	gBLUP	0.90	0.007
		Random forest	0.66	0.013
15°C	3	gBLUP	0.70	0.013
		Random forest	0.59	0.014
20°C	3	gBLUP	0.79	0.009
		Random forest	0.61	0.012

Figure S12. Performance of gBLUP and random forest in predicting F_q'/F_m' on 18 days after stratification (DAS). The F_q'/F_m' values were predicted for the test group by gBLUP (A and C) or random forest (B and D) using the GWAS-derived SNPs with $-\log_{10}$ (*P*) ≥ 3.0 identified on 18 DAS. A and B, 15°C T_{night}. C and D, 20°C T_{night}. Circles represent 59 accessions in a test set (i.e., 1/5 of the 293 accessions randomly selected as the test group). For the other two test groups, predictive performance of gBLUP models is summarized in (E). R indicates Pearson correlation coefficient and *P* is for significance. RMSE (root mean square error) has the same unit as F_q'/F_m'.

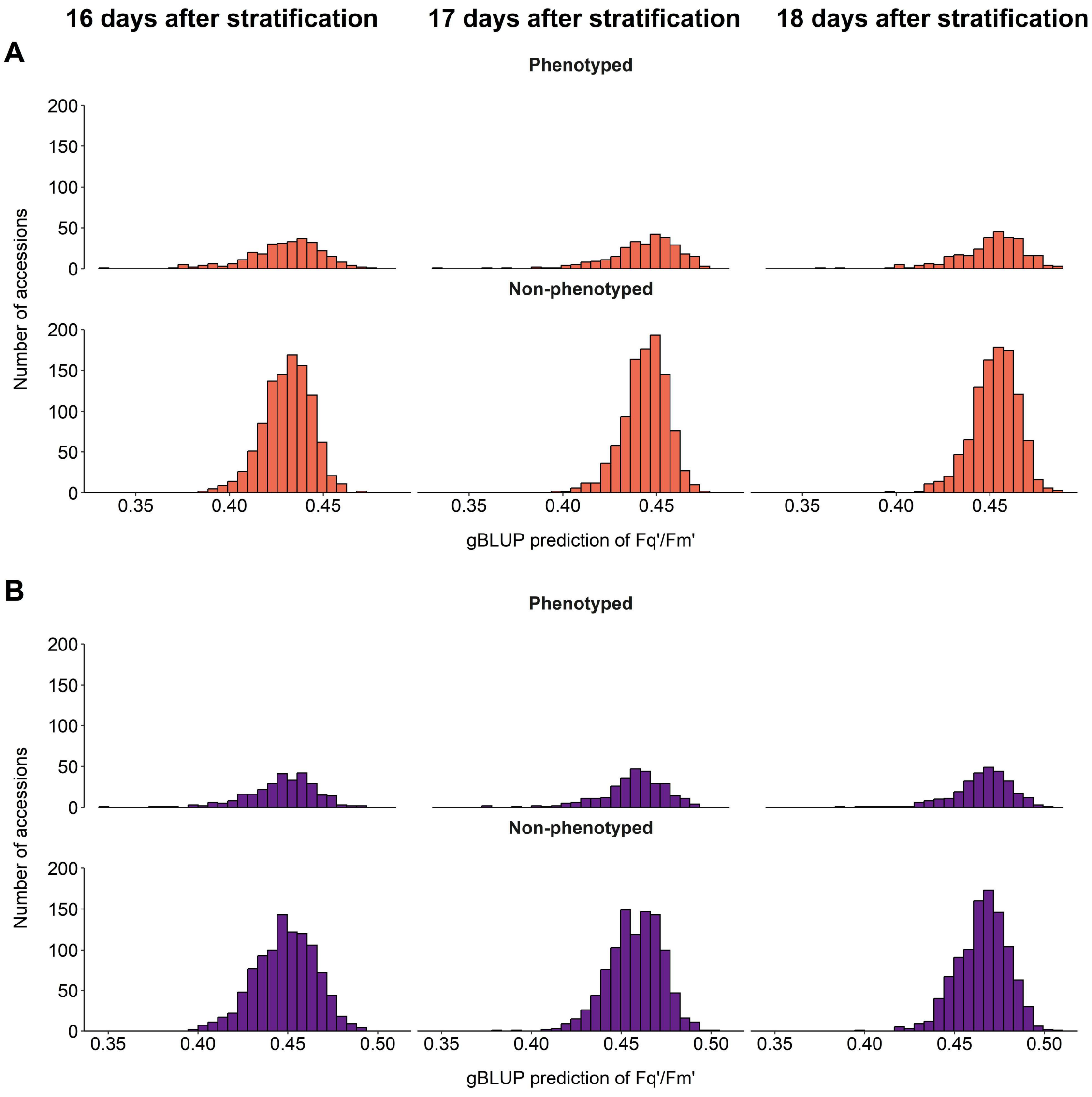

Figure S13. Distribution of predicted F_q'/F_m' in the 293 phenotyped accessions in the GWAS panel and 1,014 non-phenotyped accessions from the RegMap panel. A, 15°C T_{night}. B, 20°C T_{night}. Prediction was done by gBLUP based on the GWAS-derived SNPs with -$\log_{10}$ (*P*) ≥ 3.0 identified on 16, 17 or 18 days after stratification.

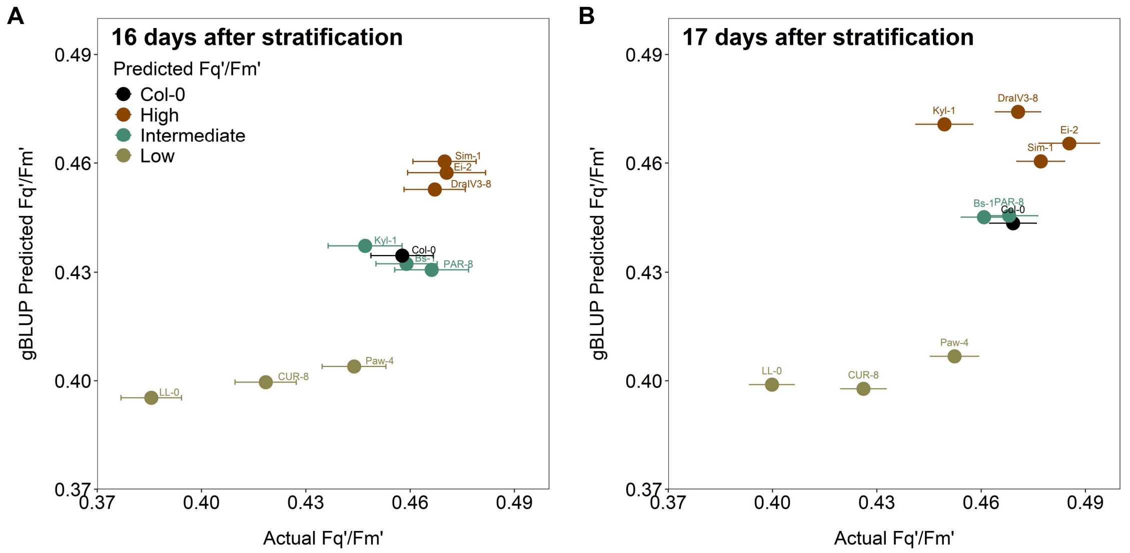

Figure S14. Experimental validation of the prediction of F_q'/F_m' by gBLUP in the 15°C T_{night} condition. Predicted and actual F_q'/F_m' on 16 (A) and 17 days after stratification (B). Prediction was based on the GWAS-derived SNPs with $-\log_{10}$ (P) $\geq$ 3.0. For validation on 18 days after stratification, see Figure 5C. Circles represent individual accessions. Different colors denote low, intermediate, and high predicted F_q'/F_m' levels. Error bars show ±SE of the adjusted entry means of actual F_q'/F_m' of individual accessions. The number of replicate plants was: Bs-1, n = 14; CUR-8, n = 19; DralIV3-8, n = 18; Ei-2, n = 3; Kyl-1, n = 4; LL-0, n = 25; PAR-8, n = 4; Paw-4, n = 11; Sim-1, n = 11; Col-0, n = 14.
